# Enhancing Patient-Dedicated Time in Clinical Encounters: A Systematic Review and Meta-analysis of Intervention Strategies

**DOI:** 10.1007/s11606-025-09843-6

**Published:** 2025-09-23

**Authors:** Clement P. Buclin, Nils Bürgisser, Amandine Berner, André Juillerat, Caroline Blanc, Matteo Coen, Pauline Darbellay Farhoumand, Violène Porto, Jessie Porzi, Jean-Luc Reny, Delphine S. Courvoisier, Thomas Agoritsas

**Affiliations:** 1 Division of General Internal Medicine, University Hospitals of Geneva, Geneva, Switzerland; 2 Faculty of Medicine, University of Geneva, Geneva, Switzerland; 3 Division of Quality of Care, University Hospitals of Geneva, Geneva, Switzerland; 4 Unit of Development and Research in Medical Education, Faculty of Medicine, University of Geneva, Geneva, Switzerland; 5 Department of Health Research Methods, Evidence, and Impact, McMaster University, Hamilton, ON Canada; 6 MAGIC Evidence Ecosystem Foundation, Oslo, Norway; 7 Division of Emergency Medicine, University Hospitals of Geneva, Geneva, Switzerland

**Keywords:** health care quality, access, and evaluation, quality indicators, health care, implementation science

## Abstract

**Background:**

Hospitals’ institutional programs designed to protect or increase the time dedicated to interactions between patients and healthcare professionals, while growing in popularity, often lack formal evaluation. This study aims to quantify the effectiveness of programs designed to protect or enhance the quality or quantity of clinical encounter time between hospitalized patients and healthcare professionals.

**Methods:**

A systematic literature review and random-effects meta-analysis were performed on Cochrane Library, Embase, and Web of Science databases. Studies had to include ≥ 80% adult inpatients in acute care, compare groups, and assess at least one of the following outcomes: patient satisfaction, length of stay, home discharge, or 30-day readmission. Screening, data extraction, and risk of bias assessment were performed independently and in duplicate. Risk of bias was assessed using the ROBINS-I tool for non-randomized trials, and the Cochrane 2.0 instrument for randomized trials.

**Results:**

A total of 117 unique studies comprising 298,517 patients were included. Compared to their controls, interventions increased the proportion of satisfied patients (+ 8% [95% CI, + 4.7 to + 11.4%]; 26 studies, 20,456 patients), the proportion of patients discharged home (+ 2.6% [95% CI, + 0.3 to + 5.0%]; 21 studies, 61,539 patients), and reduced length of stay (− 1.07 days [95% CI, − 1.62 to − 0.52]; 58 studies, 160,080 patients) without significant difference in readmission rates (− 0.8% [95% CI − 1.8 to + 0.2%]; 49 studies, 177,677 patients). Most studies were at high risk of bias, even among randomized trials. Programs varied widely in interventions, contexts, and findings.

**Discussion:**

Programs enhancing or protecting clinical encounter time in acute care may improve patient experience, care quality, and discharge processes. Higher quality randomized controlled trials evaluating such interventions are warranted. Future programs may benefit from studies that draw on multi-disciplinary knowledge and implementation sciences to identify contextual factors impacting their success.

**Systematic review registration:**

Prospero CRD42023453402.

**Supplementary Information:**

The online version contains supplementary material available at 10.1007/s11606-025-09843-6.

## BACKGROUND

Healthcare professionals’ work can be categorized into two types of tasks: clinical encounters where they interact with patients or their relatives and other tasks such as collaborative work away from the patient, evidence retrieval to answer clinical questions, or clerical duties like billing. In recent decades, time dedicated to direct patient interactions has decreased.^1,2^ For instance, only 28% of time in the emergency department^3,4^ and 23% for hospitalists^5^ are dedicated to direct patient care. Moreover, hospitalists spend between 40 and 75% of their time using computers rather than in encounters with patients.^6–8^

Several factors have been suggested to explain this shift, including the development and implementation of electronic health records,^9–11^ the increasing requirements for mandatory documentation of clinical indicators,^12,13^ or the growth of defensive medicine practices.^14,15^ Many young healthcare professionals expect more frequent and longer clinical encounters and express disappointment with the rising amount of time spent on non-clinical work, despite the recognized value of some of these tasks (e.g., documentation, evidence retrieval) for medical reasoning and decision-making.^16^ This gap between expectations and reality has been associated with lower job satisfaction,^17^ higher rates of burnout,^18,19^ and reduced patient satisfaction.^20^

A growing number of institutions have developed and implemented programs designed to protect or enhance clinical encounter time or improve its quality.^21–23^ These programs often consist of comprehensive bundles that are costly to implement and challenging to evaluate due to the complexity and variability of interventions and contexts. Many of them have not been formally assessed, despite increasing pressure to use clinical data to evaluate quality improvement projects.^24,25^ When evaluations are reported, results often vary substantially.^26,27^

This systematic review and meta-analysis aim to evaluate the effectiveness of interventions designed to protect or enhance the quality and quantity of clinical encounter time between patients and healthcare professionals. We focused on encounters in hospital acute care units and on any of the following four outcomes to provide a complementary view of effectiveness: patient satisfaction, length of stay, home discharge rates, and readmission rates.

## METHODS

We developed a protocol for the present systematic review and meta-analysis, which was registered online on Prospero no. CRD42023453402. We conducted and report our review according to the Preferred Reporting Items for Systematic Reviews and Meta-Analyses (PRISMA) checklist.^28^

### Eligibility Criteria

Eligible studies assessed interventions that aimed at increasing, protecting, or improving the quality or quantity of clinical encounter time, defined as the time during which a patient interacts with a member of the medical or nursing team. Studies had to include at least 80% of adult inpatients hospitalized in an acute care setting. Whether randomized or not, they needed to include an explicit comparator, either as before-after design or as parallel group comparison. Eligible studies assessed at least one of the following patient-centered clinical or process outcomes: (1) patient-reported experience measure (PREM) or satisfaction with care; (2) length of hospital stay; (3) discharge to patient’s home as opposed to discharge to healthcare institutions; (4) readmission before 28 or 30 days. Studies were included when an estimate and its standard error could be calculated from the data.

We excluded observational studies without intervention or those where the intervention was not primarily aimed at improving clinical encounter. We also excluded studies with less than 50 patients per arm. We anticipated that smaller studies would be unlikely to meaningfully contribute to meta-analyses, would have a higher risk of publication bias, and would more often include unrepresentative samples at baseline.^29^ We restricted eligibility to publications in English. Appendix 1 details the complete list of exclusion criteria.

### Information Sources and Search Strategy

We performed a systematic search on 21 June 2022 to identify all relevant articles. We collaborated with an experienced research librarian to devise and implement a search of EMBASE, the full Cochrane library, and Web of Science. Due to limitations in MEDLINE’s search platform, which did not support certain elements of our advanced search strategy (e.g., proximity operators like “NEXT”), we were unable to apply the same approach there and had to exclude MEDLINE from our search databases. Given the scope of our research questions and the lack of standardized search terms for the interventions of interest, we aimed for a very sensitive search, which included the three main components of our question: inpatients as the population of interest, interventions affecting clinical encounters, and clinically relevant or patient experience-related outcomes. We supplemented our search strategy by retrieving the references of excluded reviews. Appendix 2 provides our full search strategy for each database.

### Screening Process

We applied Bramer deduplication within our reference manager (EndNote™)^30^ to the final set of retrieved articles.^31^ Each title and abstracts of search records and eligible full texts were reviewed by two researchers, working independently using Covidence.^31^ Before screening, reviewers underwent training and calibration exercises to ensure sufficient agreement. Discrepancies were resolved through consensus or, when necessary, by adjudication with a third reviewer (TA).

### Data Abstraction and Categorization of Interventions

Pairs of trained reviewers extracted data from eligible trials on study characteristics (e.g., design, type of comparison), patient characteristics (e.g., age, gender), interventions characteristics (e.g., detailed description, type, duration, intensity), and outcomes of interest. Missing information was declared as missing. We extracted all available information regarding estimate of effect for each outcome, and the data to calculate their standard error. For continuous outcomes, necessary information included sample size, central tendency (mean or median), and spread (SD, IQR, or range) in both groups. Data was collected onto REDCap.^32,33^

Two authors (CPB and DC), working independently and in duplicate, thoroughly examined the description of all the interventions that were assessed and proposed broad categories of interventions. Each categorization was discussed through consensus and then reviewed by a third author (TA), to maximize reproducibility. This process was performed before any meta-analyses were conducted, and not modified afterward. It yielded the following six categories: (1) *patient education*—interventions that increase the opportunities or the quality of patient education; (2) *team change*—interventions that change the composition of healthcare teams, generally by adding a new healthcare professional to the medical rounds or patient care; (3) *nurse leadership*—interventions that include an increased shared responsibility of patient care by nursing professionals; (4) *patient oriented care*—interventions that focus on modelling care to meet patients’ needs and expectations; (5) *increased interactions*—interventions that directly increase the number of interactions between patients and healthcare professionals, such as increasing the number of rounds per day; (6) *increased continuity of care*—interventions that increase continuity of care through organizational changes, such as increasing preparedness for discharge or adding case managers. Categories were not mutually exclusive and were meant to represent the diversity of proposed changes to the organization of inpatient care. Appendix 3 displays detailed tables of the studies with their respective intervention categories.

### Assessment of Risk of Bias

Following training and calibration, all reviewers worked independently to assess risk of bias using the ROBINS-I tool for non-randomized studies. CPB and DC then adjudicated any inconsistency between reviewers and assessed bias using the Cochrane Risk of Bias 2.0 instrument for randomized trials.^34,35^ The ROBINS-I tool evaluates the risk of bias in non-randomized studies across seven domains: confounding, participant selection, intervention classification, deviations from intended intervention, missing data, outcome measurement, and selective reporting.^34^ Cochrane Risk of Bias 2.0 assesses the risk of bias across five domains: bias due to randomization (e.g., random sequence generation, allocation concealment), deviations from the intended intervention (e.g., lack of blinding with imbalances in co-interventions across arms), missing outcome data, outcome measurement, and selective reporting.^35^

### Data Synthesis and Analysis

Mean and SD were preferred when several measures were available. When means and standard deviations were not directly available, they were approximated following the Wan methodology.^36^ When only medians were available, they were considered best estimates of means and standard deviations were approximated through $\sigma =\frac{IQR}{1.35}$, where IQR is the interquartile range. When articles presented only medians and range, the means were approximated through $\chi=\frac{min+2\ast\widetilde x+max}4$ where *χ* is the mean, min is the minimum, x͂ is the median, and max is the maximum. The SD were approximated using $\frac{max-min}{2*Qnorm(\frac{n-0.375}{n+0.25})}$ where Qnorm is the quantile function with a standard deviation of 1 and means of 0. For categorical outcomes, necessary information included sample size and either a number, a risk, or a percentage of patients presenting the outcome, in both groups. For satisfaction questionnaire, Likert scales and similar categorical answers were dichotomized in “Satisfied” regrouping answers reporting fully or mostly satisfied patients and “Unsatisfied” regrouping all remaining answers. For satisfaction questionnaires that reported results on different numeric scales, we standardized the difference in means by the average of the variability in the intervention and control groups, using the formula $\frac{Meanintervention-Meancontrol}{(SDcontrol+SDintervention)/2}$. This approach allowed us to account for differences in scale ranges and ensure comparability across satisfaction measures.

When possible, for each category of interventions and outcomes, we performed frequentist, random-effects meta-analysis given the expected heterogeneity of interventions and contexts. We summarized heterogeneity using the *I*^2^ statistic and interpreted an *I*^2^ value of 0 to 40% as heterogeneity that might not be important, 30 to 60% as moderate, 50 to 90% as substantial, and 75 to 100% as considerable heterogeneity.^37^ We tested for publication bias by visually inspecting funnel plots for each outcome (Appendix 4).

Finally, we also examined studies according to study design, and type of intervention. Length of stay was additionally stratified by type of estimates (mean versus median) and length of stay prior to intervention. Due to the low number of adjusted analyses and the high variability of adjustment variables between studies, meta-analyses of adjusted outcomes are only reported as exploratory sensitivity analyses.

## RESULTS

### Search Results

**Figure 1:**
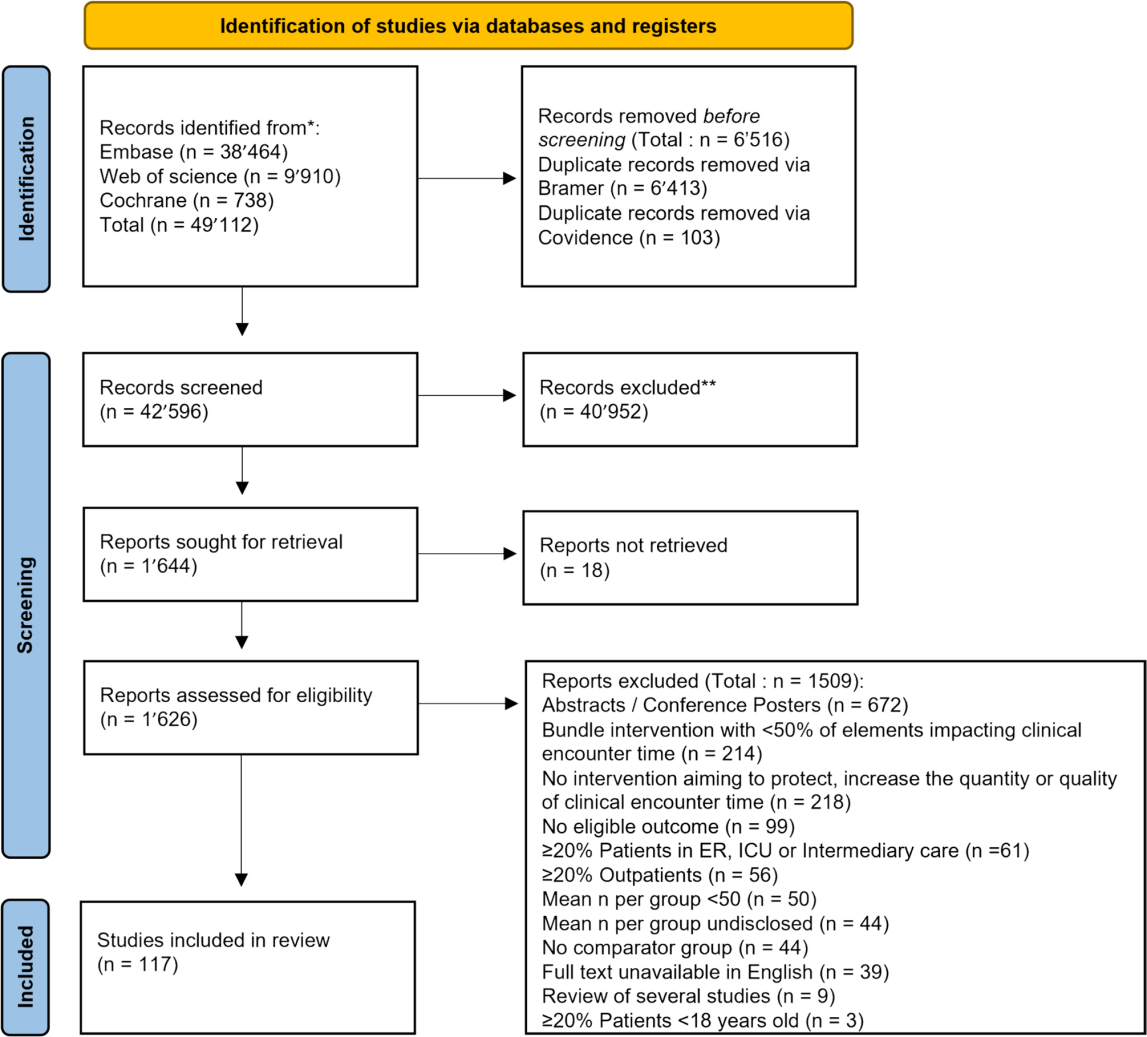
Preferred Reporting Items for Systematic Review and Meta-Analyses flow diagram for identification of relevant studies for inclusion.

Of the 42,596 citations retrieved, 117 articles, representing 298,517 patients, met the eligibility criteria and were included. Figure 1 presents study selection and Appendix 3 the list of all included articles.

### Study Characteristics

Studies were published between 1990 and 2022, with a median sample size of 484 participants. Included patients were, on average, 64.2 years old, and 54% were women. Table 1 presents trial and patient characteristics.

**Table 1:** Included Studies Characteristics

		*N* = 117
Publication year (median [IQR])		2017 [2013, 2020]
Median number of included patients [IQR]		484 [193, 2000]
Design (*n* (%))		
	Non-randomized	93 (79.5)
	Randomized	24 (20.5)*
Type of comparator (*n* (%))		
	Before-after	62 (53.0)
	Parallel	48 (41.0)
	Difference-in-difference	7 (6.0)
Study Setting (*n* (%))		
	Africa	1 (0.9)
	Asia	21 (17.9)
	Europe	23 (19.7)
	North America	66 (56.4)
	Oceania	6 (5.1)
Intervention categories (*n* (%))**		
	Patient education	25 (21.4)
	Team change	54 (45.3)
	Nurse leadership	10 (8.5)
	Patient oriented care	24 (20.5)
	Increased interactions	60 (51.3)
	Increased continuity of care	36 (30.8)
Women in control group (mean (SD))		54.0 (17.4)^‡^
Women in intervention group (mean (SD))		53.2 (17.4)^‡^
Age in control group (mean)		65.08^§^
Age in intervention group (mean)		63.35^§^

^*^Included 10 (41.7%) cluster randomized control trials (CRCTs)

^**^Categories were not mutually exclusive and were meant to represent the diversity of proposed changes to the organization of inpatient care (see the “Data Abstraction and Categorization of Interventions” section)

^‡^Included 25.6% of missing data

^§^Included 26.5% of missing data and was often reported categorized or without SD, which precluded computation of an overall SD

### Summary of Findings

Table 2 summarizes the pooled effects of intervention strategies to enhance patient-dedicated time in clinical encounter on four chosen outcomes: patient experience or satisfaction with care; length of hospital stay; readmission rates; home discharge. We provide meta-analytical estimates across all interventions, but also within each of the six intervention categories.

**Table 2:** Pooled Effects of Intervention Strategies to Enhance Patient-dedicated Time in Clinical Encounter

			***N*** **studies**	***N*** **patients**	**Estimate**	**95% CI**	***p*-value**	***I***^**2**^
***Patient satisfaction*** (Dichotomized proportion of satisfied patients in %)*					***Risk difference***			
	**All interventions**		**26**	**20,456**	** + 8.0%**	**[+ 4.7%: + 11.4%]**	** < *0.001***	***79.3%***
		Patient education	7	8465	+ 11.7%	[+ 1.0%: + 22.4%]	*0.031*	*82.4%*
		Team change	9	16,450	+ 5.0%	[− 0.4%: + 10.5%]	*0.072*	*89.3%*
		Nurse leadership	1	310	+ 8.8%	[− 0.6%: + 18.1%]	*0.066*	*–*
		Patient oriented care	9	2176	+ 13.3%	[+ 3.3%: + 23.3%]	*0.009*	*82.9%*
		Increased interaction	15	5253	+ 7.0%	[+ 3.7%: + 10.3%]	< *0.001*	*61.9%*
		Increased continuity	10	7999	+ 6.2%	[+ 1.4%: + 10.9%]	*0.012*	*79.8%*
***Patient satisfaction*** (Difference in standardized mean)**					***Mean difference***			
	**All interventions**		**17**	**3807**	** + 0.86**	**[+ 0.22: + 1.49]**	***0.008***	***99.9%***
		Patient education	3	614	+ 0.67	[− 0.46: + 1.8]	*0.247*	*98.3%*
		Team change	3	899	+ 0.67	[− 0.85: + 3.36]	*0.243*	*100.0%*
		Nurse leadership	0	0	–	–	*–*	*–*
		Patient oriented care	8	1687	+ 0.56	[− 0.09: + 1.21]	*0.09*	*98.4%*
		Increased interaction	6	1174	+ 0.92	[− 0.37: + 2.22]	*0.161*	*99.6%*
		Increased continuity	4	784	+ 1.24	[− 0.45: + 2.93]	*0.149*	*99.8%*
***Length of hospital stay**** (mean in days)*					***Mean difference***			
	**All interventions**		**58**	**160,080**	− **1.07**	**[**− **1.62:** − **0.52]**	** < *0.001***	***100.0%***
		Patient education	12	18,069	− 2.27	[− 3.88: − 0.67]	*0.006*	*99.8%*
		Team change	29	54,351	− 1.06	[− 1.66: − 0.45]	*0.001*	*98.2%*
		Nurse leadership	6	8986	− 1.18	[− 4.33: + 1.97]	*0.463*	*94.0%*
		Patient oriented care	12	22,752	− 1.49	[− 2.85: − 0.13]	*0.032*	*99.0%*
		Increased interaction	33	132,470	− 0.70	[− 1.37: − 0.04]	*0.039*	*100.0%*
		Increased continuity	14	64,153	− 1.46	[− 2.58: − 0.34]	*0.01*	*100.0%*
***Home discharge rate***					***Risk difference***			
	**All interventions**		**21**	**61,539**	** + 2.6%**	**[+ 0.3%: + 5.0%]**	***0.03***	***87.3%***
		Patient education	3	5289	+ 0.9%	[− 1.8%: + 3.7%]	*0.517*	*0.0%*
		Team change	14	27,603	+ 2.0%	[− 0.5%: + 4.5%]	*0.122*	*64.2%*
		Nurse leadership	1	175	+ 1.7%	[− 9.1%: + 12.6%]	*0.754*	*–*
		Patient oriented care	1	248	+ 8.2%	[+ 0.9%: + 15.5%]	*0.028*	*–*
		Increased interaction	9	52,567	+ 2.7%	[− 0.6%: + 6.0%]	*0.107*	*93.4%*
		Increased continuity	6	41,678	+ 0.2%	[− 1.9%: + 2.4%]	*0.828*	*70.5%*
***30-days readmission rate***					***Risk difference***			
	**All interventions**		**49**	**177,677**	− **0.8%**	**[**− **1.8%: + 0.2%]**	***0.110***	***83.2%***
		Patient education	8	17,099	− 5.2%	[− 7.9%: − 2.6%]	< *0.001*	*79.2%*
		Team change	28	61,229	− 0.3%	[− 1.7%: + 1.0%]	*0.624*	*85.2%*
		Nurse leadership	4	23,725	1.1%	[− 0.1%: + 2.2%]	*0.063*	*5.7%*
		Patient oriented care	3	8884	− 3.5%	[− 10.9%: + 3.9%]	*0.357*	*92.4%*
		Increased interaction	27	109,145	− 0.8%	[− 1.9%: + 0.2%]	*0.102*	*64.3%*
		Increased continuity	18	99,161	− 0.5%	[− 1.3%: + 0.2%]	*0.173*	*48.9%*

^*^Proportion of satisfied patients represents the percentage classified as “satisfied” according to each study’s original scale, including only those reporting being fully or mostly satisfied

^**^Difference in standardized mean represents the difference in mean satisfaction scores between groups, standardized by the standard deviations of the groups, to allow comparison across studies using satisfaction scales with varied ranges (e.g., 0–5, 0–7, 0–100…)

#### Patient Satisfaction

Of the 48 studies on patient satisfaction, 43 reported all required data. Twenty-six (24,263 patients) reported proportions and 17 (3807 patients) reported means (detailed flow chart in Appendix 5).

Clinical encounter protection programs were associated with an increase in both the proportion of patients satisfied with their hospitalization (+ 8.0% [+ 4.7%: + 11.4%]), and the standardized mean levels of satisfaction (+ 0.86 [+ 0.22: + 1.49]). This effect was consistent across study designs and interventions categories, although it did not reach significance in several categories with a smaller number of studies. The largest effect was observed in “Patient oriented care” interventions (9 studies, 2176 patients) with an increase of + 13.3% [+ 3.3%: + 23.3%] (Table 2).

The 6 studies (3490 patients) that adjusted their analyses for patient-level factors—typically adjusting for age, sex, ethnicity, and previous health status—reported a non-significant decrease in patient satisfaction (odds ratio, 0.48 [0.13:1.82] *I*^2^ = 90%, mean differences of − 1.38 [− 6.51: + 3.75] *I*^2^ = 44%.

#### Length of Stay

Of the 100 studies reporting length of stay (LOS), 58 provided all required data to calculate an unadjusted mean difference, representing 160,080 patients (detailed flow chart in Appendix 5). There was a substantial heterogeneity in the observed mean difference, with individual studies’ findings ranging from a decrease of − 8.5 days [− 11.05: − 5.95] to an increase of + 13.9 days [− 0.62: + 28.42].

Overall, clinical encounter protection programs were associated with a statistically significant reduction of − 1.07 [− 1.62: − 0.52] in average LOS. This effect was strongest for before-after studies − 1.22 [− 1.88: − 0.55] and smaller and non-significant for parallel studies − 0.78 [− 1.95: + 0.40] and difference-in-difference studies − 0.88 [− 2.64: + 0.88] (Forest plots in Appendix 6). Study design also influenced the observed effect, with non-randomized studies showing a mean LOS difference of − 1.18 [− 1.74: − 0.62], while no significant effect was found in RCTs and cluster RCTs, which had a mean difference of − 0.32 [− 2.25: + 1.60].

Pooled LOS effects were relatively similar across all intervention categories except patient education, which showed a larger reduction of − 2.3 days. Mean difference in LOS increased with baseline LOS from − 0.4 days [− 0.7: − 0.1] for a baseline LOS < 5 days to − 2.1 days [− 3.7: − 0.5] for a baseline LOS > 10 days (Appendix 6). The 4 studies (18,047 patients) that adjusted their analyses for patient-level factors—typically adjusting for age and previous health status—reported a smaller but still significant reduction of LOS of − 0.36 days [− 0.54: − 0.19], with very small residual heterogeneity *I*^2^ = 0%.

#### Rate of Home Discharge

A total of 21 studies reported home discharge rate, encompassing 61,539 patients. The observed risk difference in home discharge rates ranged from − 5.2% [− 14.5%: + 4.1%] to + 16.9% [+ 8.8%: + 24.9%] (detailed flow chart in Appendix 5).

Home discharges were more frequent in the clinical encounter protection programs (risk difference + 2.6% [+ 0.3%: + 5.0%]). Results were consistent across study design and intervention categories, although failed to reach significance in several categories with smaller number of studies. None of the included articles presented adjusted analyses.

#### 30-Day Readmission Rate

Of the 60 studies reporting readmission rates, 49 reported readmissions at about one month (i.e., 28 or 30 days) in each group, representing 177,677 patients (detailed flow chart in Appendix 5). The risk difference in readmission rate ranged from − 12.0% [− 21.4%: − 2.7%] to + 9.2% [+ 1.1%: + 17.3%].

Overall, clinical encounter protection programs were not associated with a significantly change in readmission rates, regardless of study design or baseline readmission rates. The rates were also similar across all intervention categories except patient education, which decreased the risk by − 5.2% [− 7.9%: − 2.6%] (Table 2). Adjusted analyses (conducted in 7 eligible studies representing 15,956 patients) were consistent with an absence of effect (pooled OR 0.97 [0.87:1.08], *I*^2^ = 44.8%).

#### Risk of Bias

**Figure 2:**
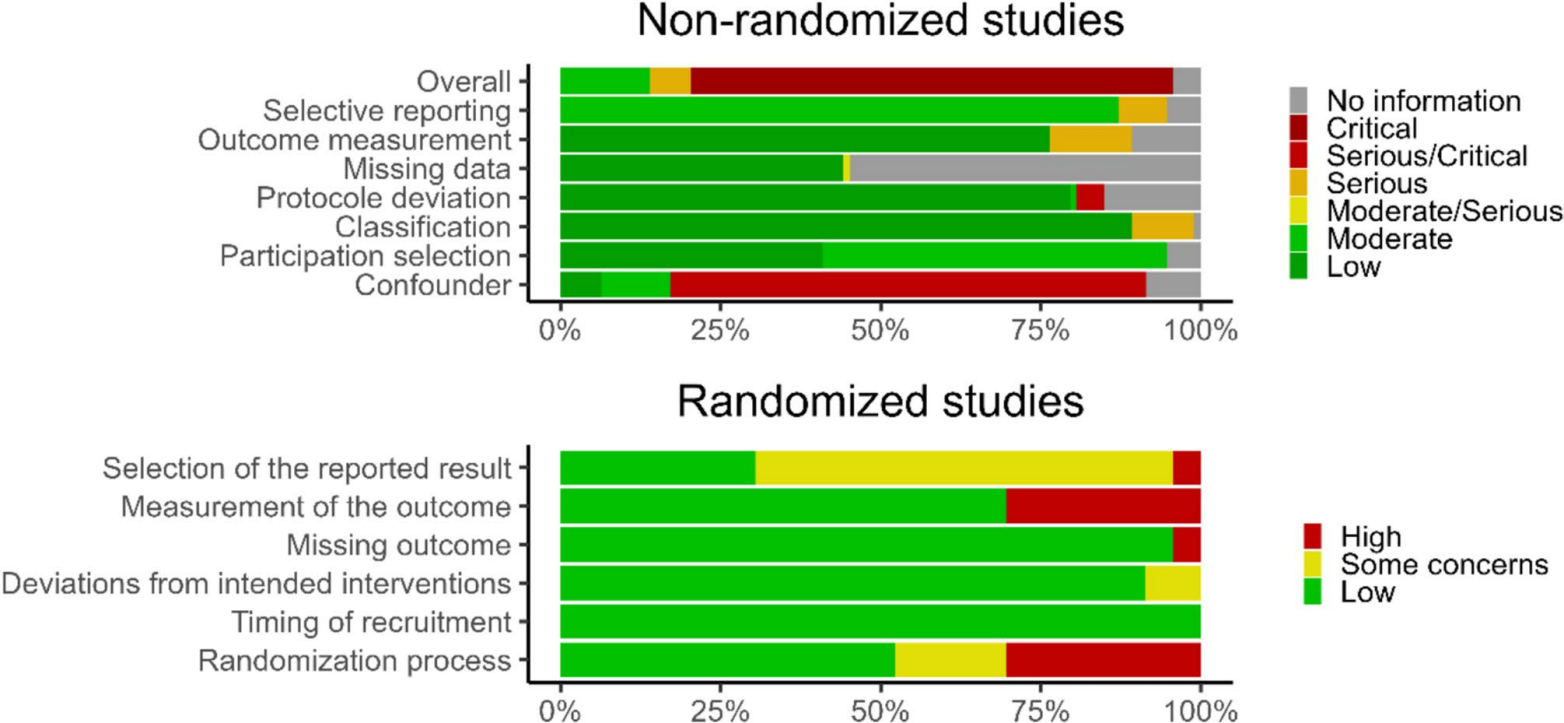
Risk of bias for non-randomized studies (top panel, *n* = 93) and randomized trials (bottom panel, *n* = 24) studies.

Risk of bias were evaluated by outcome of interest (full descriptive tables for each domain available, Appendix 7). Overall, most studies were at high risk of bias, even among randomized controlled trials (Fig. 2). Furthermore, funnel plots (Appendix 4) suggested that there could be some publication bias for the LOS outcome.

## Discussion

Our large systematic review of intervention strategies aiming to enhance the quality or quantity of patient-dedicated time in clinical encounter included nearly 300,000 patients mainly from hospitals in North America, Europe, and Asia. Overall, the tested programs may increase patient satisfaction (+ 8% of satisfied patients) and the proportion of patients discharged home (+ 2.6%), reduce length of stay (− 1 day), with no significant impact on readmission rates. Programs varied greatly in their interventions and implementation contexts, resulting in important heterogeneity of observed outcomes, and the risk of bias remained high, even in randomized studies. Nevertheless, some results were robust enough to study the design and type of comparison, particularly the substantial and consistent effect on patient satisfaction across all subgroups examined.

The main strength of this study is the scope and comprehensiveness of our search, which provides the first systematic summary of the current body of evidence on quality improvement programs aimed at enhancing clinical encounters with patients. Our search, screening, evidence synthesis, and appraisal followed a rigorous methodology.

The main limitation lies in the quality of the eligible studies, presenting a high risk of bias and a fair likelihood of publication bias. Effect sizes for all our clinical outcomes might be affected by publication and small study bias, particularly for length of stay, which likely lacks publications showing increased LOS. This issue reflects the lack of rigorous evaluation of interventions that many institutions tend to implement as part of internal quality improvement programs without official scientific evaluation. Most included studies are non-randomized and do not adjust for potential confounders, failing to follow clear international reporting guidelines.^38^ Although we did not formally apply GRADE overall appraisal of the evidence, the certainty of the evidence for all outcomes is bound to be low to very low due to the risk of bias, publication bias, imprecision of the effect, and inconsistency in findings. Before-after studies systematically showed larger effects on clinical outcomes than parallel studies, likely due to a higher risk of temporal confounding. This is partially confirmed by the consistency of higher effects reported in observational studies compared to RCTs and cluster RCTs.

Applicability is also limited by the lack of representation of African and South American hospitals, who were not represented in the articles retrieved by our search.

Another limitation of this study is how this body of evidence rarely reported on costs—direct and indirect—needed for the implementation of their interventions. This is a critical gap given the current economic constraint on healthcare systems and the heavy burden imposed on healthcare workers. These programs have the potential to improve the quality of care, but they need to be designed and adapted locally within a given system or institution, requiring significant human and financial resources. Their impact on healthcare professionals already facing substantial demands also needs to be investigated to ensure the programs’ sustainability beyond initial implementation, as well as their possible impact on job satisfaction.^39,40^

Included interventions were mostly bundles that aimed at increasing the quality—rather than the quantity per se—of patient-dedicated time in clinical encounters. The observed impact may thus be mediated by creating a set of conditions to allocate time more efficiently and to better meet patients’ needs and expectations, rather than by a simple increase in time dedicated to clinical encounters. Experts have already pointed out simple practices to enhance clinical encounters, for instance, prepare with intention, listen intently and completely, agree on what matters most for the patient, connect with the patient’s story, and explore emotional cues.^41^ Effectiveness—possibly also cost-effectiveness—of quality improvement programs may result from the capacity to generate better conditions for the daily provision of kind and careful care and unhurried conversations. ^42,43^

## Conclusion

In conclusion, we have summarized the current body of evidence on programs to enhance and protect patient-dedicated time in clinical encounters found several positive associations of these programs with patient satisfaction, home discharge, and length of stay. Future programs designed to enhance clinical encounters should be prospectively assessed, with well-designed and conducted studies, ideally randomizing allocation. Methods need to build on multidisciplinary knowledge from implementation sciences to shed light on contextual factors enabling or hindering their success.^44–48^ Resources, cost-effectiveness, and sustainability need to be more thoroughly assessed. The impact on patients but also on healthcare professionals and their capacity to provide kind and careful care are avenues for research. The further development of Artificial Intelligence and Large Language Models may offer new tools for healthcare professionals, such as automated documentation, which could free up some of the time currently dedicated to clerical tasks.^49^ Such efforts are needed to create the best value-added time for clinical encounters and ensure that this freed time is used to replace patients care at the center of hospital time. 

## Supplementary Information

Below is the link to the electronic supplementary material.

Supplementary file 1 (DOCX 1.83 MB)

Supplementary file 1 (XLSX 50.1 KB)

## Data Availability

The complete dataset used for this review as well as the data collection forms are available in supplementary materials. The R code is available from corresponding author upon request.

## Declarations:

### Patient and Public Involvement:

We did not include patients and public.
