# Supplementary material for: Enhancing Patient-Dedicated Time in Clinical Encounters: A Systematic Review and Meta-analysis of Intervention Strategies
Source: J Gen Intern Med. 2025 Sep 23;41(1):197–205. doi: 10.1007/s11606-025-09843-6 (PMC12855675; doi:10.1007/s11606-025-09843-6)
Supplement: Supplementary file 1 — (DOCX 1.83 MB) [file 11606_2025_9843_MOESM1_ESM.docx]

# Appendix 1: Inclusion and exclusion criteria

Table S1 Inclusion and exclusion criteria

| Criteria type | Inclusion criteria | Exclusion criteria |
| --- | --- | --- |
| Study characteristics | Is an article presenting a single study with full text available in English.  **An article** is defined as any published text that presents its own data and is the complete version of the text.  **It includes** original articles, brief reports or letters.  **It excludes** abstracts, posters and any published text that is not the complete version of a text. | - Not an article (e.g., poster, conference abstract, ...) - Full text not in English - Review of several studies (literature review, whether systematic or narrative) |
| Population | A majority (≥80%) of adult inpatient **excluding** inpatients taken care in intensive care units, intermediate care units and emergency rooms. Group size $\geq$ 50 patients per group.  **NB:** In the specific case where an article has a population that doesn’t fit the criteria but presents an analysis stratified by subgroups it should be included. | - <80% adults - <80% inpatients - Inpatients mostly hospitalized in Intensive or intermediate care units, or emergency rooms - <50 patients per groups |
| Intervention | Intervention or bundle of interventions that includes at least 50% of elements that aim to either   - **Protect** - **Increase the quantity** - **Increase the quality**   of clinical encounter time.  **Clinical encounter time** is defined as a time of interaction between the patient (and/or the patient family caregiver) and a medical or nursing health care professional.  **Medical or nursing health care professionals** include licensed doctors, nurses and all variant of assistant nurses. Other healthcare professionals may be present and impacted by the intervention but shouldn't be the only professionals involved. | - No intervention that aims to protect, increase the quantity or the quality of clinical encounter time between medical professionals and patients. - Intervention occurs in the absence of either patients or eligible health care professionals (ex: huddle outside the patient room, consultant that doesn't visit the patient, intervention during operating time = patient asleep, ...) - <50% of the elements of the bundle intervention target the clinical encounter time   **This excludes**: Nurse, assistant nurse and doctor students. |
| Comparison | A comparator group must be present.  Comparators can be :   - a comparison in time (before/after, including difference-in-difference, time-series) - a *parallel* comparison at the same time (exposure/control)   **Time series**: are a type of time comparison that compares data measured at several time points over a period before and after an intervention (interruption). | - No comparator group   **This exludes:** comparator that aren’t groups included in the study but general comparator like a country or a hospital’s average. |
| Outcomes | At least 1 numerical outcome that is either:   1. Related to patient satisfaction/patient experience 2. A clinical outcome (complications rates, length of stay, early readmissions rates, ...) 3. A process-related outcomes (outcome evaluating the intrinsic success of the intervention)   Process outcomes encompass the measure of the intrinsic success of the process of an intervention. A good example would be the number of interruptions of the medical round in an intervention aiming to lower the number of interruptions. | - No eligible outcome - No numerical outcome   **Those exclude:** Only financial outcomes, Medical Professional satisfaction.  **Those exclude**: self-assessment of outcomes like abilities or attitude self-reported questionnaires |

# Appendix 2: Search Strategy

## EMBASE

('hospital patient'/exp OR 'intensive care'/exp OR 'hospital':ab,ti OR 'inpatient*':ab,ti OR 'in$patient*':ab,ti OR 'medical center*':ab,ti OR ((surgical NEAR/2 (unit OR center*)):ab,ti) OR 'hospitali$ed patient*':ab,ti OR 'in$hospital patient*':ab,ti OR 'icu':ab,ti OR 'care unit*':ab,ti OR ((emergency NEAR/2 (department* OR service* OR nursing)):ab,ti) OR ((intensive NEXT/2 (unit OR care)):ab,ti)) AND ((((time OR duration OR length*) NEAR/3 ('hourly round*' OR 'attending round*' OR 'medical scribe*' OR 'direct patient care' OR 'lean round*' OR 'round*' OR encounter* OR 'per patient')):ab,ti) OR (('time spent' NEXT/2 patient*):ab,ti) OR ((('doctor*' OR 'physician*' OR 'nurse*' OR 'patient*') NEAR/2 (relationship OR encounter OR round* OR bedside)):ab,ti) OR 'bundle of care':ab,ti OR 'ward task':ab,ti OR 'value adding care':ab,ti OR 'patient care':ab,ti OR 'interprofessional round*':ab,ti OR 'process improvement':ab,ti OR 'multidisciplinary round*':ab,ti OR 'direct patient care':ab,ti OR 'care model based intervention*':ab,ti OR 'quality improvement program*':ab,ti OR 'discharge planning program*':ab,ti OR 'rounding model*':ab,ti OR 'rounds report':ab,ti OR ((care NEXT/1 (plan* OR polic* OR model*)):ab,ti) OR 'multi-component intervention*':ab,ti OR ((implementation NEAR/2 (project OR program* OR method)):ab,ti)) AND ('treatment duration'/de OR 'hospital discharge'/de OR 'treatment outcome'/de OR 'clinical outcome'/de OR 'length of stay'/de OR 'patient satisfaction'/de OR 'hospital stays':ab,ti OR 'length of stay':ab,ti OR 'treatment duration':ab,ti OR 'enhancing care':ab,ti OR 'los':ab,ti OR 'improvement*':ab,ti OR (((treatment OR clinical) NEAR/2 (outcome* OR efficacy OR effectiveness)):ab,ti) OR 'readmission rate*':ab,ti OR 'earl* discharge':ab,ti OR 'discharge time':ab,ti OR ((patient NEXT/1 (satisfaction OR need* OR experience* OR communication OR feedback OR recovery OR expectation*)):ab,ti) OR ((quality NEXT/2 (life OR improvement OR care)):ab,ti))

## Cochrane

https://www.cochranelibrary.com/advanced-search/search-manager

(inpatient*:ab,ti OR "medical center*":ab,ti OR ((surgical NEAR/2 (unit OR center*)):ab,ti) OR "hospitalised patient*":ab,ti OR "hospitalized patient*":ab,ti OR "inhospital patient*":ab,ti OR "icu":ab,ti OR "care unit*":ab,ti OR ((emergency NEAR/2 (department* OR service* OR nursing)):ab,ti) OR ((intensive NEXT/2 (unit OR care)):ab,ti)) AND ((((time OR duration OR length*) NEAR/3 ("hourly round*" OR "attending round*" OR "medical scribe*" OR "direct patient care" OR "lean round*" OR round* OR encounter* OR "per patient")):ab,ti) OR (("time spent" NEXT/2 patient*):ab,ti) OR (((doctor* OR physician* OR nurse* OR patient*) NEAR/2 (relationship OR encounter OR round* OR bedside)):ab,ti) OR "bundle care":ab,ti OR "ward task":ab,ti OR "value adding care":ab,ti OR "patient care":ab,ti OR "interprofessional round*":ab,ti OR "process improvement":ab,ti OR "multidisciplinary round*":ab,ti OR "direct patient care":ab,ti OR "care model based intervention*":ab,ti OR "quality improvement program*":ab,ti OR "discharge planning program*":ab,ti OR "rounding model*":ab,ti OR "rounds report":ab,ti OR ((care NEXT/1 (plan* OR polic* OR model*)):ab,ti) OR "multi-component intervention*":ab,ti OR ((implementation NEAR/2 (project OR program* OR method)):ab,ti)) AND ("hospital stays":ab,ti OR "length stay":ab,ti OR "treatment duration":ab,ti OR "enhancing care":ab,ti OR "los":ab,ti OR (((treatment OR clinical) NEAR/2 (outcome* OR efficacy OR effectiveness)):ab,ti) OR "readmission rate*":ab,ti OR "earl* discharge":ab,ti OR "discharge time":ab,ti OR ((patient NEXT/1 (satisfaction OR need* OR experience* OR communication OR feedback OR recovery OR expectation*)):ab,ti) OR (quality NEXT/2 (life OR improvement OR care)):ab,ti)

## WOS

https://www.webofscience.com/wos/woscc/advanced-search

TS=(("inpatient*" OR "medical center*" OR ("surgical" NEAR/2 ("unit" OR "center*")) OR "hospitalised patient*" OR "hospitalized patient*"OR "inhospital patient*" OR "icu" OR "care unit*" OR ("emergency" NEAR/2 ("department*" OR "service*" OR "nursing")) OR ("intensive" NEAR/2 ("unit" OR "care"))) AND (((time OR duration OR length*) NEAR/3 ("hourly round*" OR "attending round*" OR "medical scribe*" OR "direct patient care" OR "lean round*" OR round* OR encounter* OR "per patient")) OR ("time spent" NEAR/2 patient*) OR ((doctor* OR physician* OR nurse* OR patient*) NEAR/2 (relationship OR encounter OR round* OR bedside)) OR "bundle care" OR "ward task" OR "value adding care" OR "patient care" OR "interprofessional round*" OR "process improvement" OR "multidisciplinary round*" OR "direct patient care" OR "care model based intervention*" OR "quality improvement program*" OR "discharge planning program*" OR "rounding model*" OR "rounds report" OR (care NEAR/1 (plan* OR polic* OR model*)) OR "multi-component intervention*" OR (implementation NEAR/2 (project OR program* OR method))) AND ("hospital stays" OR "length stay" OR "treatment duration" OR "enhancing care" OR "los" OR ((treatment OR clinical) NEAR/2 (outcome* OR efficacy OR effectiveness)) OR "readmission rate*" OR "earl* discharge" OR "discharge time" OR (patient NEAR/1 (satisfaction OR need* OR experience* OR communication OR feedback OR recovery OR expectation*)) OR (quality NEAR/2 (life OR improvement OR care))))

# Appendix 3: Included studies and their assigned interventions

Table S2 Articles included in meta-analysis

| **Article Label** | **First Author** | **Title** | **Journal** | **Publication Year** |
| --- | --- | --- | --- | --- |
| Ahmad 2011 | Ahmad, A. | The impact of twice-daily consultant ward rounds on the length of stay in two general medical wards | Clin Med J R Coll Phys Lond | 2011 |
| Ahmad 2015 | Ahmad, A. | A cost-benefit analysis of twice-daily consultant ward rounds and clinical input on investigation and pharmacy costs in a major teaching hospital in the UK | BMJ Open | 2015 |
| Ahrens 2013 | Ahrens, S. L. | Using evidence to improve satisfaction with medication side-effects education on a Neuro-medical surgical unit | J Neurosci Nurs | 2013 |
| Aicher 2019 | Aicher, B. O. | Reduced length of stay and 30-day readmission rate on an inpatient vascular surgery service | J Vasc Nurs | 2019 |
| Allerby 2020 | Allerby, K. | Striving for a more person-centered psychosis care: results of a hospital-based multi-professional educational intervention | BMC Psychiatry | 2020 |
| Anandan 2022 | Anandan, S. | A Retrospective Study Analyzing a Palliative Care-Hospital Medicine Collaboration to Improve Quality of Care of Patients With Advanced Illness | Am J Hosp Palliat Care | 2022 |
| Anderson 2017 | Anderson, M. E. | Geriatric Hip Fracture Care: Fixing a Fragmented System | Perm J | 2017 |
| Arbaje 2010 | Arbaje, A. I. | The geriatric floating interdisciplinary transition team | J Am Geriatr Soc | 2010 |
| Ayaad 2019 | Ayaad, O. | Impact of Structured Nurse Leader Rounds on Satisfaction With Nursing Care Among Patients With Cancer | J Nurs Scholarsh | 2019 |
| Batsis 2007 | Batsis, J. A. | Effects of a hospitalist care model on mortality of elderly patients with hip fractures | J Hosp Med | 2007 |
| Begue 2012 | Begue, A. | Retrospective study of multidisciplinary rounding on a thoracic surgical oncology unit | Clin J Oncol Nurs | 2012 |
| Ben-Menachem 1996 | Ben-Menachem, T. | Balancing service and education: Improving internal medicine residencies in the managed care era | Am J Med | 1996 |
| Bhattacharyya 2013 | Bhattacharyya, R. | A unique orthogeriatric model: A step forward in improving the quality of care for hip fracture patients | Int J Surg | 2013 |
| Blood 2019 | Blood, T. D. | Assessment of a Geriatric Hip Fracture Program: Analysis of Harmful Adverse Events Using the Global Trigger Tool | J Bone Jt Surg Am Vol | 2019 |
| Braude 2017 | Braude, P. | Evaluation and establishment of a ward-based geriatric liaison service for older urological surgical patients: Proactive care of Older People undergoing Surgery (POPS)-Urology | BJU Int | 2017 |
| Brennan 2019 | Brennan, M. J. | An acute care for elders quality improvement program for complex, high-cost patients yields savings for the system | J Hosp Med | 2019 |
| Brosey 2015 | Brosey, L. A. | Effectiveness of structured hourly nurse rounding on patient satisfaction and clinical outcomes | J Nurs Care Qual | 2015 |
| Chava 2019 | Chava, R. | Multidisciplinary rounds in prevention of 30-day readmissions and decreasing length of stay in heart failure patients: A community hospital based retrospective study | Medicine (Baltimore) | 2019 |
| Chen 2022 | Chen, J. | Effect of Clinical Nursing Pathway Intervention Based on Evidence-Based Medicine on Venous Thrombosis in Long-Term Bedridden Patients | J Healthc Eng | 2022 |
| Christensen 2017 | Christensen, K. | Evaluation of a standardized ward round in a prenatal inpatient setting | Int J Gynaecol Obstet | 2017 |
| Coates 2021 | Coates, E. | Focusing hospitalist roles on either admitting or rounding facilitates unit-based assignment and is associated with improved discharge efficiency | Hosp Pract | 2021 |
| Courtenay 2007 | Courtenay, M. | An evaluation of a specialist nurse prescriber on diabetes in-patient service delivery | Pract Diabetes Int | 2007 |
| Courtright 2019 | Courtright, K. R. | Electronic Health Record Mortality Prediction Model for Targeted Palliative Care Among Hospitalized Medical Patients: a Pilot Quasi-experimental Study | J Gen Intern Med | 2019 |
| Cowan 2006 | Cowan, M. J. | The effect of a multidisciplinary hospitalist/physician and advanced practice nurse collaboration on hospital costs | J Nurs Adm | 2006 |
| Curley 1998 | Curley, C. | A firm trial of interdisciplinary rounds on the inpatient medical wards: an intervention designed using continuous quality improvement | Med Care | 1998 |
| Cyrus 2022 | Cyrus, R. M. | Effect of an Attending Nurse on Timeliness of Discharge, Patient Satisfaction, and Readmission | J Nurs Manag | 2022 |
| Córcoles-Jiménez 2021 | Córcoles-Jiménez, M. P. | Effectiveness of a best practice guideline to manage pain in surgical patients | Appl Nurs Res | 2021 |
| DePesa 2020 | DePesa, C. D. | A multidisciplinary approach to decreasing length of stay in acute care surgery patients | J Adv Nurs | 2020 |
| Donovan 2020 | Donovan, A. K. | The impact of residents sitting at the bedside on patient satisfaction during team rounds | Patient Educ Couns | 2020 |
| Dunn 2017 | Dunn, A. S. | The Impact of Bedside Interdisciplinary Rounds on Length of Stay and Complications | J Hosp Med | 2017 |
| El Baz 2009 | El Baz, N. | Coronary artery bypass graft (CABG) surgery patients in a clinical pathway gained less in health-related quality of life as compared with patients who undergo CABG in a conventional-care plan | J Eval Clin Pract | 2009 |
| Finn 2018 | Finn, K. M. | Effect of increased inpatient attending physician supervision on medical errors, patient safety, and resident education: A randomized clinical trial | JAMA Intern Med | 2018 |
| Gandsas 2007 | Gandsas, A. | Robotic Telepresence: Profit Analysis in Reducing Length of Stay after Laparoscopic Gastric Bypass | J Am Coll Surg | 2007 |
| Gilfillan 2016 | Gilfillan, C. | A 7-day team-based model of care in general medicine: implementation and outcomes at 12 months | INTERNAL MEDICINE JOURNAL | 2016 |
| Gormley 2019 | Gormley, D. K. | Impact of Nurse-Led Interprofessional Rounding on Patient Experience | Nurs Clin North Am | 2019 |
| Griffiths 1996 | Griffiths, P. | Clinical outcomes for nurse-led in-patient care | Nurs Times | 1996 |
| Griffiths 2001 | Griffiths, P. | Substitution of a nursing-led inpatient unit for acute services: randomized controlled trial of outcomes and cost of nursing-led intermediate care | Age and ageing | 2001 |
| Guan 2021 | Guan, H. | The influence of integrated healthcare on the nursing effect, negative emotions, and heart failure indicators in acute heart failure patients | Am J Transl Res | 2021 |
| Gutierrez 2021 | Gutierrez, J. | Implementing a Telehospitalist Program Between Veterans Health Administration Hospitals: Outcomes, Acceptance, and Barriers to Implementation | J Hosp Med | 2021 |
| Hafner 2021 | Hafner, T. | Care of Geriatric Patients with Lumbar Spine, Pelvic, and Acetabular Fractures before and after Certification as a Geriatric Trauma Center DGUÂ®: A Retrospective Cohort Study | Medicina (Kaunas) | 2021 |
| Halvachizadeh 2021 | Halvachizadeh, S. | The effect of geriatric comanagement (GC) in geriatric trauma patients treated in a level 1 trauma setting: A comparison of data before and after the implementation of a certified geriatric trauma center | PLoS ONE | 2021 |
| Hefti 2017 | Hefti, E. | Hospital consumer assessment of healthcare providers and systems scores relating to pain following the incorporation of clinical pharmacists into patient education prior to joint replacement surgery | Pharm Pract | 2017 |
| Heyzer 2021 | Heyzer, L. | Integrated Hip Fracture Care Pathway (IHFCP): reducing complications and improving outcomes | Singapore Med J | 2021 |
| Hock Lee 2011 | Hock Lee, K. | Bringing generalists into the hospital: Outcomes of a family medicine hospitalist model in Singapore | J Hosp Med | 2011 |
| Hu 2016 | Hu, W. | Improving doctor-patient communication: Content validity examination of a novel urinary system-simulating physical model | Patient Preference Adherence | 2016 |
| Hu 2020 | Hu, R. | The effects of a transitional care program on discharge readiness, transitional care quality, health services utilization and satisfaction among Chinese kidney transplant recipients: A randomized controlled trial | Int J Nurs Stud | 2020 |
| Iberti 2016 | Iberti, C. T. | Hospitalist-vascular surgery comanagement: effects on complications and mortality | Hosp Pract | 2016 |
| Jakobsson 2020 | Jakobsson, S. | Patient safety before and after implementing person-centred inpatient care - A quasi-experimental study | Journal of clinical nursing | 2020 |
| Javed 2021 | Javed, A. | Improved Clinical Competence and Patients Satisfaction: Comparison of Patient-centered, Interdisciplinary Collaborative Round versus Traditional Post-partum Round | J Coll Phys Surg Pak | 2021 |
| Kielty 2000 | Kielty, M. | Quality pain management in a community hospital using limited resources | Dis Manage | 2000 |
| Klaiber 2018 | Klaiber, U. | Impact of preoperative patient education on the prevention of postoperative complications after major visceral surgery: The cluster randomized controlled PEDUCAT trial | Trials | 2018 |
| Krantz 2004 | Krantz, M. J. | Impact of a cardiac risk reduction program in vulnerable patients hospitalized with coronary artery disease | Pharmacotherapy | 2004 |
| Krepper 2014 | Krepper, R. | Evaluation of a Standardized Hourly Rounding Process (SHaRP) | JOURNAL FOR HEALTHCARE QUALITY | 2014 |
| Kripalani 2019 | Kripalani, S. | A transition care coordinator model reduces hospital readmissions and costs | Contemp Clin Trials | 2019 |
| Kullberg 2019 | Kullberg, A. | Improved patient satisfaction 2 years after introducing person-centred handover in an oncological inpatient care setting | J Clin Nurs | 2019 |
| Lai 2021 | Lai, Y. F. | General medicine at the frontline of acute care delivery: Comparison with family medicine hospitalist model implementation in Singapore | Proc Singapore Healthcare | 2021 |
| Lai 2022 | Lai, Y. F. | One-Bed-One-Team-Does an Integrated General Hospital Inpatient Model Improve Care Outcomes and Productivity: An Observational Study | Front Public Health | 2022 |
| Law 2016 | Law, C. | Impact of specialized inpatient IBD care on outcomes of IBD hospitalizations: A cohort study | Gastroenterology | 2016 |
| Lee 2021 | Lee, J. C. | Impact of an orthogeriatric collaborative care model for older adults with hip fracture in a community hospital setting | Can J Surg | 2021 |
| Lofgren 1990 | Lofgren, R. P. | Post-call transfer of resident responsibility: Its effect on patient care | J Gen Intern Med | 1990 |
| Löfgren 2015 | Löfgren, S. | Power to the patient: care tracks and empowerment a recipe for improving rehabilitation for hip fracture patients | Scand J Caring Sci | 2015 |
| Malfait 2020 | Malfait, S. | The impact of bedside handovers on relevant clinical indicators: A matched-controlled multicenter longitudinal study | J Adv Nurs | 2020 |
| Maniaci 2020 | Maniaci, M. J. | Goal-Directed Achievement Through Geographic Location (GAGL) Reduces Patient Length of Stay and Adverse Events | Am J Med Qual | 2020 |
| Manzano 2019 | Manzano, J. G. | Demonstrating value: association of cost and quality outcomes with implementation of a value-driven oncology-hospitalist inpatient collaboration for patients with lung cancer | BMJ Open Qual | 2019 |
| Mao 2020 | Mao, G. | The use of hospital consumer assessment of healthcare services and the Press Ganey medical practice surveys in guiding surgical patient care practices | Surg Neurol Intl | 2020 |
| Martin 1998 | Martin, D. P. | Randomized trial of a patient-centered hospital unit | Patient Educ Couns | 1998 |
| McNicholas 2017 | McNicholas, A. | Improving Patient Experience Through Nursing Satisfaction | J Trauma Nurs | 2017 |
| Meehan 2016 | Meehan, A. | Health System Quality Improvement: Impact of Prompt Nutrition Care on Patient Outcomes and Health Care Costs | J Nurs Care Qual | 2016 |
| Meijuan Yang 2018 | Meijuan Yang, G. | Pilot Study of a Palliative Care and Medical Oncology Co-Rounding Model for Advanced Cancer Inpatients in a Tertiary Hospital in Singapore | J Palliative Med | 2018 |
| Merel 2016 | Merel, S. E. | Sitting at patients' bedsides may improve patients' perceptions of physician communication skills | J Hosp Med | 2016 |
| Monash 2017 | Monash, B. | Standardized Attending Rounds to Improve the Patient Experience: A Pragmatic Cluster Randomized Controlled Trial | J Hosp Med | 2017 |
| Mudge 2006 | Mudge, A. | Controlled trial of multidisciplinary care teams for acutely ill medical inpatients: enhanced multidisciplinary care | INTERNAL MEDICINE JOURNAL | 2006 |
| Mudge 2012 | Mudge, A. M. | Improving hospital outcomes in patients admitted from residential aged care: Results from a controlled trial | Age Ageing | 2012 |
| Mudge 2013 | Mudge, A. M. | Improving quality of delirium care in a general medical service with established interdisciplinary care: a controlled trial | INTERNAL MEDICINE JOURNAL | 2013 |
| Mulugeta 2020 | Mulugeta, H. | The effect of hourly nursing rounds on patient satisfaction at Debre Markos Referral Hospital, Northwest Ethiopia: A non-randomized controlled clinical trial | Int J Afr Nurs Sci | 2020 |
| Negarandeh 2014 | Negarandeh, R. | Impact of regular nursing rounds on patient satisfaction with nursing care | Asian Nurs Res | 2014 |
| O'Connor 2011 | O'Connor, A. B. | Restructuring an inpatient resident service to improve outcomes for residents, students, and patients | Acad Med | 2011 |
| O'Leary 2016 | O'Leary, K. J. | Effect of patient-centred bedside rounds on hospitalised patients' decision control, activation and satisfaction with care | BMJ Qual Saf | 2016 |
| Ogawa 2019 | Ogawa, A. | Quality of care in hospitalized cancer patients before and after implementation of a systematic prevention program for delirium: the DELTA exploratory trial | Supportive Care Cancer | 2019 |
| Okere 2016 | Okere, A. N. | Comparison of a pharmacist-hospitalist collaborative model of inpatient care with multidisciplinary rounds in achieving quality measures | Am J Health-Syst Pharm | 2016 |
| Oldham 2021 | Oldham, M. A. | Proactive Integration of Mental Health Care in Hospital Medicine: PRIME Medicine | JOURNAL OF THE ACADEMY OF CONSULTATION-LIAISON PSYCHIATRY | 2021 |
| Palmer Jr 2001 | Palmer Jr, H. C. | The effect of a hospitalist service with nurse discharge planner on patient care in an academic teaching hospital | Am J Med | 2001 |
| Rajasekaran 2015 | Rajasekaran, K. | Development of a Quality Care Plan to Reduce Otolaryngologic Readmissions | Otolaryngol Head Neck Surg | 2015 |
| Ramirez 2016 | Ramirez, J. | Patient Satisfaction with Bedside Teaching Rounds Compared with Nonbedside Rounds | SOUTHERN MEDICAL JOURNAL | 2016 |
| Ritchie 2017 | Ritchie, R. | Impact of an educational training program on restorative care practice of nursing assistants working with hospitalized older patients | J Clin Outcomes Manage | 2017 |
| Roberts 2016 | Roberts, R. P. | Early Compared with Delayed Physician Rounds on Patient Satisfaction of Postpartum Women | Obstet Gynecol | 2016 |
| Rohatgi 2018 | Rohatgi, N. | Surgical Comanagement by Hospitalists in Colorectal Surgery | J Am Coll Surg | 2018 |
| Rubin 2005 | Rubin, A. S. | Effects on processes and costs of care associated with the addition of an internist to an inpatient psychiatry team | Psychiatr Serv | 2005 |
| Sand-Jecklin 2014 | Sand-Jecklin, K. | A quantitative assessment of patient and nurse outcomes of bedside nursing report implementation | J Clin Nurs | 2014 |
| Shin 2018 | Shin, N. | The Effect of Intentional Nursing Rounds Based on the Care Model on Patients' Perceived Nursing Quality and their Satisfaction with Nursing Services | Asian Nurs Res | 2018 |
| Shirreff 2019 | Shirreff, L. | Introduction of Physician-Nurse Bedside Rounding and Ward Task List to Improve Quality of Care in Gynaecology: Prospective, Single-Blinded, Pre- and Post-Intervention Study | J Obstet Gynaecol Can | 2019 |
| Singh 2012 | Singh, S. Lipscomb G. | Daily consultant gastroenterologist ward rounds: Reduced length of stay and improved inpatient mortality | Frontline Gastroenterol | 2012 |
| Singh 2012 | Singh, S. Tamira S. | Impact of localizing general medical teams to a single nursing unit | Journal of hospital medicine | 2012 |
| Sivaram 1997 | Sivaram, C. A. | Introducing case management to a general medicine ward team of a teaching hospital | Acad Med | 1997 |
| Sledge 2015 | Sledge, W. H. | Multidisciplinary proactive psychiatric consultation service: Impact on length of stay for medical inpatients | Psychother Psychosom | 2015 |
| Solberg 2013 | Solberg, L. M. | A quality improvement program to increase nurses' detection of delirium on an acute medical unit | GERIATRIC NURSING | 2013 |
| Soric 2016 | Soric, M. M. | Economic and patient satisfaction outcomes of a layered learning model in a small community hospital | Am J Health-Syst Pharm | 2016 |
| Southey 2014 | Southey, D. | Continuity of care by cardiothoracic nurse practitioners: Impact on outcome | Asian Cardiovasc Thorac Ann | 2014 |
| Southwick 2014 | Southwick, F. | Applying athletic principles to medical rounds to improve teaching and patient care | Acad Med | 2014 |
| Spellberg 2012 | Spellberg, B. | A controlled investigation of optimal internal medicine ward team structure at a teaching hospital | PLoS ONE | 2012 |
| Sriram 2017 | Sriram, K. | A comprehensive nutrition-focused quality improvement program reduces 30-day readmissions and length of stay in hospitalized patients | J Parenter Enter Nutr | 2017 |
| Steiner 2001 | Steiner, A. | Therapeutic nursing or unblocking beds? A randomised controlled trial of a post-acute intermediate care unit | BRITISH MEDICAL JOURNAL | 2001 |
| Sunkara 2020 | Sunkara, P. R. | Impact of structured interdisciplinary bedside rounding on patient outcomes at a large academic health centre | BMJ Qual Saf | 2020 |
| Tadros 2015 | Tadros, R. O. | The effect of a hospitalist comanagement service on vascular surgery inpatients | J Vasc Surg | 2015 |
| Van Groningen 2022 | Van Groningen, N. | 'Physician advocates': a novel strategy for improving the value of hospital care by employing hospitalists part time to support non-hospitalist physicians | Hosp Pract | 2022 |
| Van Grootven 2021 | Van Grootven, B. | Geriatric co-management for cardiology patients in the hospital: A quasi-experimental study | J Am Geriatr Soc | 2021 |
| Wahbi-Izzettin 2018 | Wahbi-Izzettin, O. | United we stand, divided we conquer: pilot study of multidisciplinary General Medicine Heart Failure Care Program | Intern Med J | 2018 |
| Wang 2022 | Wang, H. | EFFECT of COMPREHENSIVE NURSING MODEL on the BAD MOOD and QUALITY of LIFE of ELDERLY PATIENTS with CORONARY HEART DISEASE | Acta Med Mediterr | 2022 |
| Wollenhaup 2017 | Wollenhaup, C. A. | Implementation of a Modified Bedside Handoff for a Postpartum Unit | JOURNAL OF NURSING ADMINISTRATION | 2017 |
| Wong 2021 | Wong, A. P. Y. | Feasibility & Efficacy of Deprescribing rounds in a Singapore rehabilitative hospital- a randomised controlled trial | BMC Geriatr | 2021 |
| Wood 2016 | Wood, J. G. | Collaborative Care on the Stroke Unit: A Cross-Sectional Outcomes Study | J Neurosci Nurs | 2016 |
| Xu 2020 | Xu, J. | Application of inter-professional care model in patients with aneurysmal subarachnoid haemorrhage | J Nurs Manag | 2020 |
| Yang 2021 | Yang, G. M. | Comparing the effect of a consult model versus an integrated palliative care and medical oncology co-rounding model on health care utilization in an acute hospital - an open-label stepped-wedge cluster-randomized trial | Palliative Med | 2021 |
| Yee 2022 | Yee, D. K. H. | Orthogeriatric Multidisciplinary Co-Management Across Acute and Rehabilitation Care Improves Length of Stay, Functional Outcomes and Complications in Geriatric Hip Fracture Patients | Geriatr Orthop Surg Rehabit | 2022 |
| Zhang 2021 | Zhang, L. | Effect of a nursing intervention strategy oriented by Orem's self-care theory on the recovery of gastrointestinal function in patients after colon cancer surgery | Am J Transl Res | 2021 |
| Zhu 2021 | Zhu, T. | Orem's self-care to treat acute coronary syndrome after PCI helps improve rehabilitation efficacy and quality of life | Am J Transl Res | 2021 |
| de Las Casas 2021 | de Las Casas, R. | Establishing a perioperative medicine for older people undergoing surgery service for general surgical patients at a district general hospital | Clin Med J R Coll Phys Lond | 2021 |

Table S3 Categorisation of interventions

| Proposed theme | N of articles | Articles selected for the theme |
| --- | --- | --- |
| Patient education | 25 | Ahrens 2013, Aicher 2019, Ayaad 2019, Chava 2019, Chen 2022, Christensen 2017, El Baz 2009, Gilfillan 2016, Guan 2021, Heyzer 2021, Hu 2016, Hu 2020, Kielty 2000, Klaiber 2018, Krantz 2004, Kripalani 2019, Löfgren 2015, Martin 1998, Mudge 2013, Rajasekaran 2015, Soric 2016, Sriram 2017, Wahbi-Izzettin 2018, Zhang 2021, Zhu 2021 |
| Team change | 53 | Aicher 2019, Anderson 2017, Arbaje 2010, Batsis 2007, Begue 2012, Bhattacharyya 2013, Blood 2019, Braude 2017, Brennan 2019, Chava 2019, Cowan 2006, Curley 1998, Cyrus 2022, de Las Casas 2021, Finn 2018, Gilfillan 2016, Gutierrez 2021, Hafner 2021, Halvachizadeh 2021, Hefti 2017, Heyzer 2021, Hock Lee 2011, Iberti 2016, Javed 2021, Lai 2021, Law 2016, Lee 2021, Lofgren 1990, Löfgren 2015, Manzano 2019, Mao 2020, Meijuan Yang 2018, Mudge 2006, O'Connor 2011, Okere 2016, Palmer Jr 2001, Ritchie 2017, Rohatgi 2018, Rubin 2005, Sivaram 1997, Sledge 2015, Soric 2016, Southey 2014, Southwick 2014, Spellberg 2012, Sunkara 2020, Tadros 2015, Van Grootven 2021, Wong 2021, Wood 2016, Xu 2020, Yang 2021, Yee 2022 |
| Nurse leadership | 10 | Courtenay 2007, Gormley 2019, Griffiths 1996, Griffiths 2001, Gutierrez 2021, Lai 2022, Löfgren 2015, Mudge 2012, Southey 2014, Steiner 2001 |
| Patient oriented care | 24 | Ahrens 2013, Allerby 2020, Anandan 2022, Ayaad 2019, Brennan 2019, Christensen 2017, Donovan 2020, Dunn 2017, Gormley 2019, Jakobsson 2020, Klaiber 2018, Kripalani 2019, Kullberg 2019, Löfgren 2015, Malfait 2020, Martin 1998, Merel 2016, O'Leary 2016, Roberts 2016, Shin 2018, Shirreff 2019, Wang 2022, Zhang 2021, Zhu 2021 |
| Increased interactions | 60 | Ahmad 2011, Ahmad 2015, Aicher 2019, Anandan 2022, Ben-Menachem 1996, Blood 2019, Braude 2017, Brennan 2019, Brosey 2015, Chava 2019, Chen 2022, Coates 2021, Córcoles-Jiménez 2021, Courtenay 2007, Courtright 2019, Cowan 2006, Curley 1998, de Las Casas 2021, DePesa 2020, Dunn 2017, Gandsas 2007, Gilfillan 2016, Gormley 2019, Guan 2021, Hafner 2021, Kielty 2000, Krepper 2014, Kullberg 2019, Malfait 2020, McNicholas 2017, Meehan 2016, Meijuan Yang 2018, Monash 2017, Mudge 2013, Mulugeta 2020, Negarandeh 2014, O'Connor 2011, O'Leary 2016, Ogawa 2019, Okere 2016, Oldham 2021, Rajasekaran 2015, Ramirez 2016, Ritchie 2017, Rubin 2005, Sand-Jecklin 2014, Shirreff 2019, Singh Lipscomb 2012, Sivaram 1997, Sledge 2015, Solberg 2013, Spellberg 2012, Sriram 2017, Sunkara 2020, Van Groningen 2022, Van Grootven 2021, Wollenhaup 2017, Wong 2021, Wood 2016, Xu 2020 |
| Increased continuity of care | 36 | Ahmad 2011, Ahmad 2015, Aicher 2019, Anderson 2017, Arbaje 2010, Begue 2012, Coates 2021, Cyrus 2022, Dunn 2017, El Baz 2009, Gandsas 2007, Gilfillan 2016, Gormley 2019, Guan 2021, Hock Lee 2011, Javed 2021, Krepper 2014, Lai 2022, Maniaci 2020, McNicholas 2017, Monash 2017, Mudge 2006, Mudge 2012, O'Connor 2011, Palmer Jr 2001, Rajasekaran 2015, Rubin 2005, Shin 2018, Shirreff 2019, Singh Lipscomb 2012, Singh Tamira 2012, Sivaram 1997, Southwick 2014, Van Grootven 2021, Wahbi-Izzettin 2018, Yang 2021 |

# Appendix 4: Funnel plots by outcomes


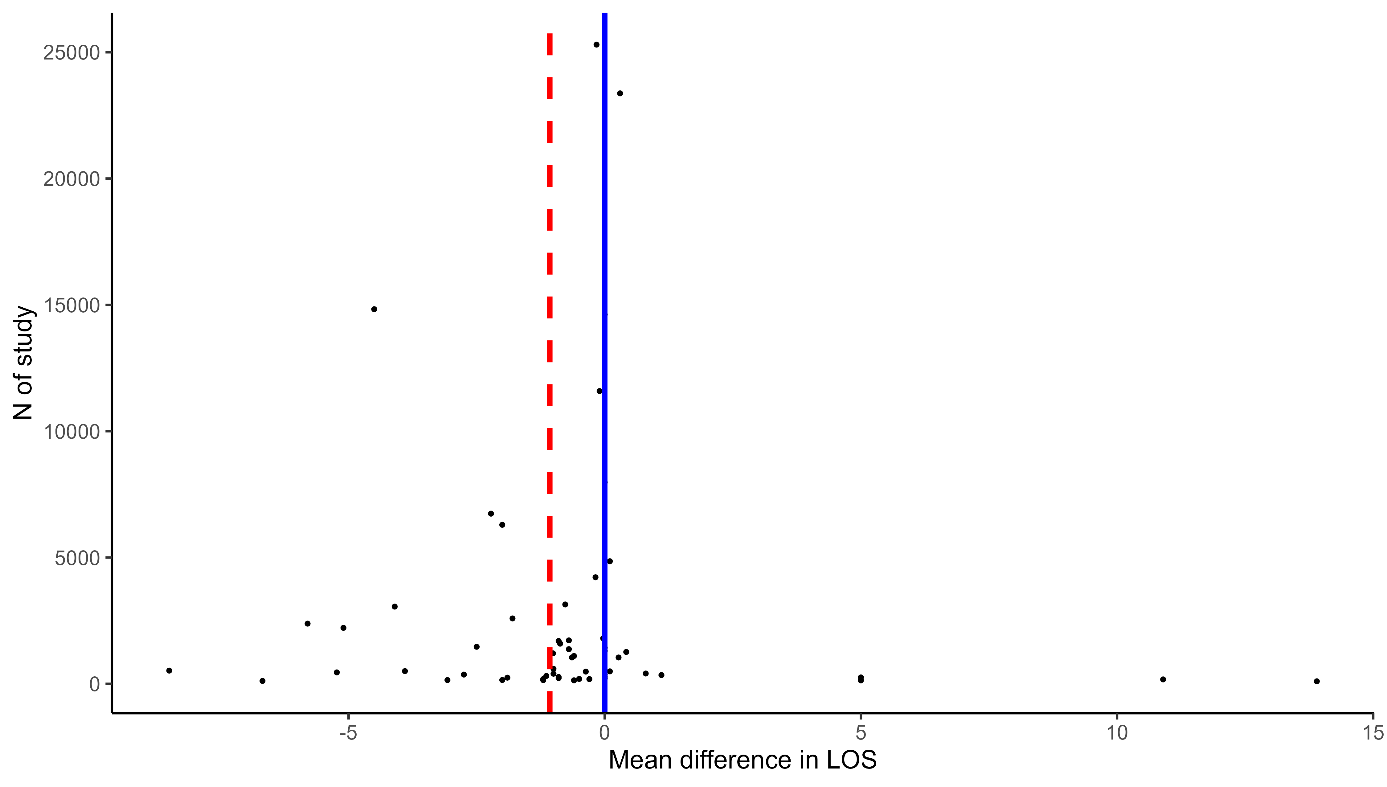


Figure S2: Funnel plot for length of stay. Red dotted line: Random effect estimate, blue line: mean difference of 0


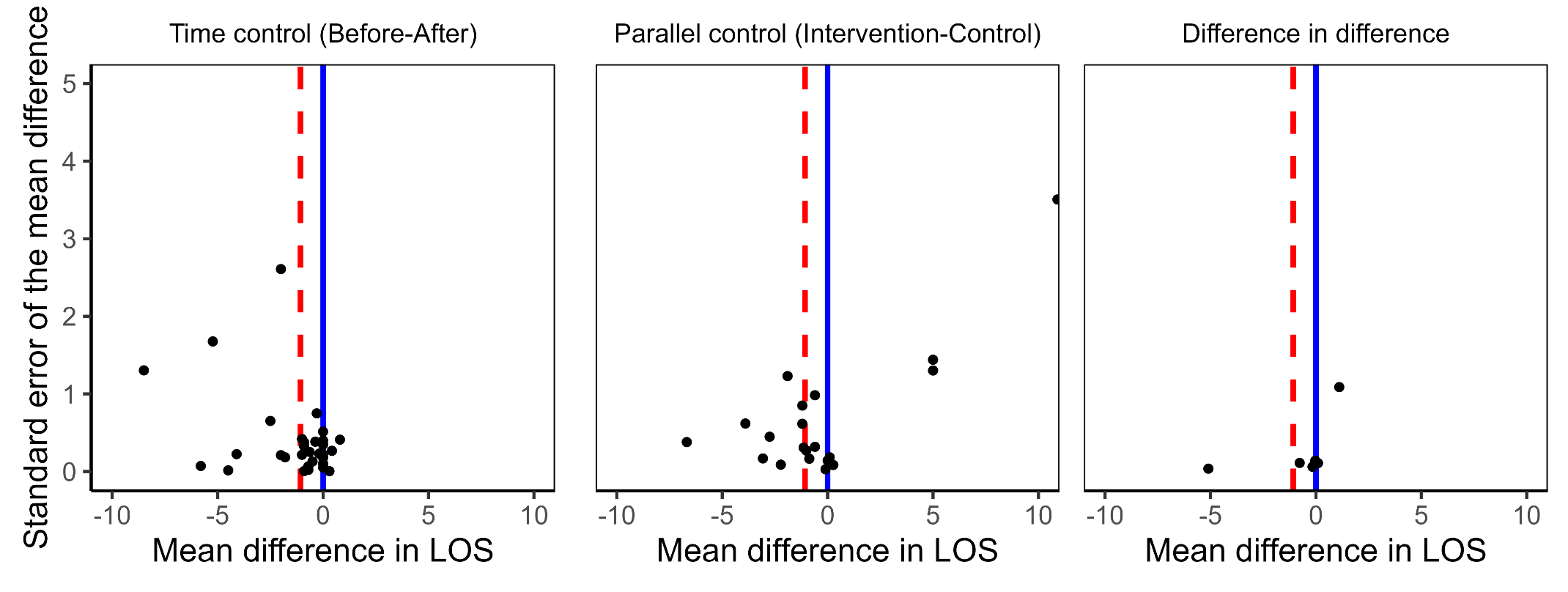


Figure S3: Funnel plot for length of stay by study designs. Red dotted line: Random effect estimate, blue line: mean difference of 0


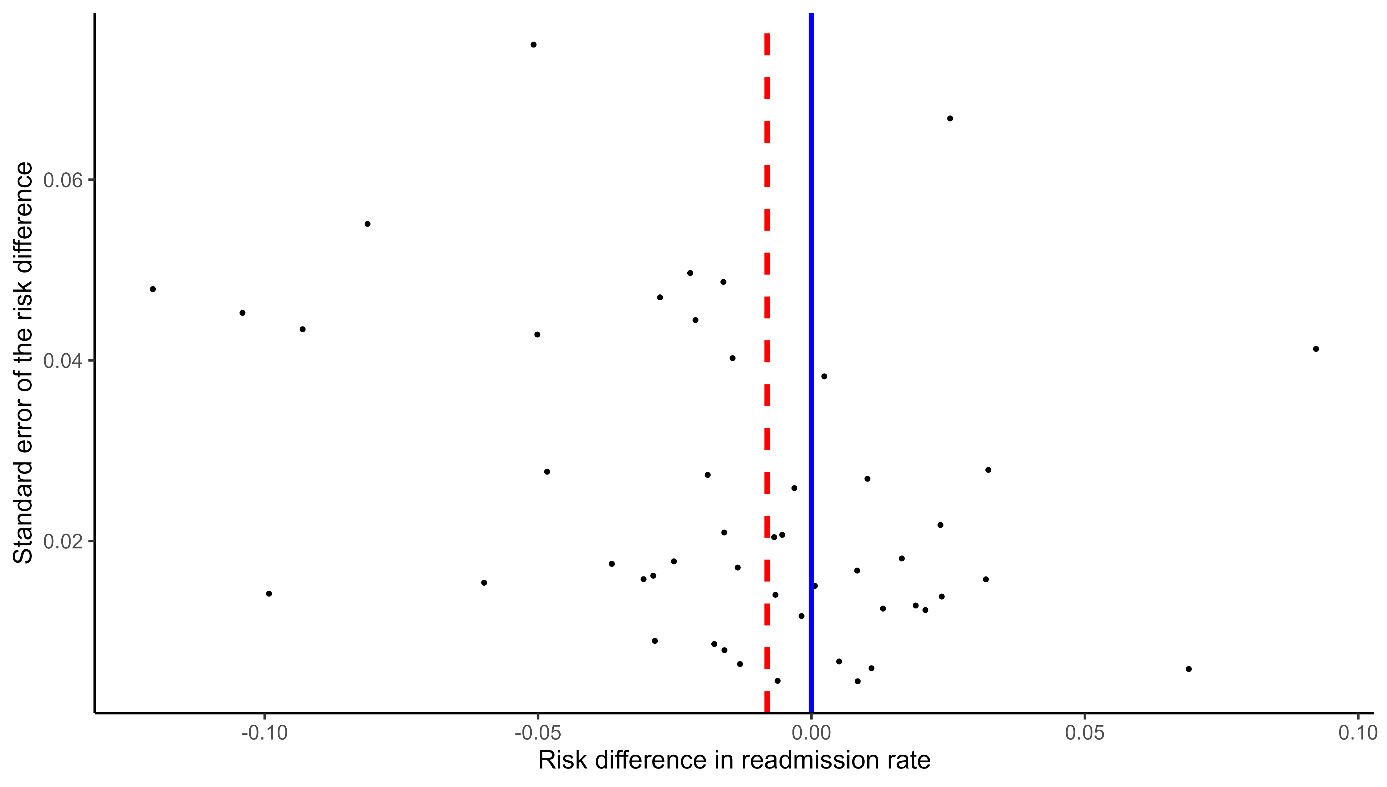


*Figure S4: Funnel plot for readmission rate. Red dotted line: Random effect estimate, blue line: risk difference of 0*


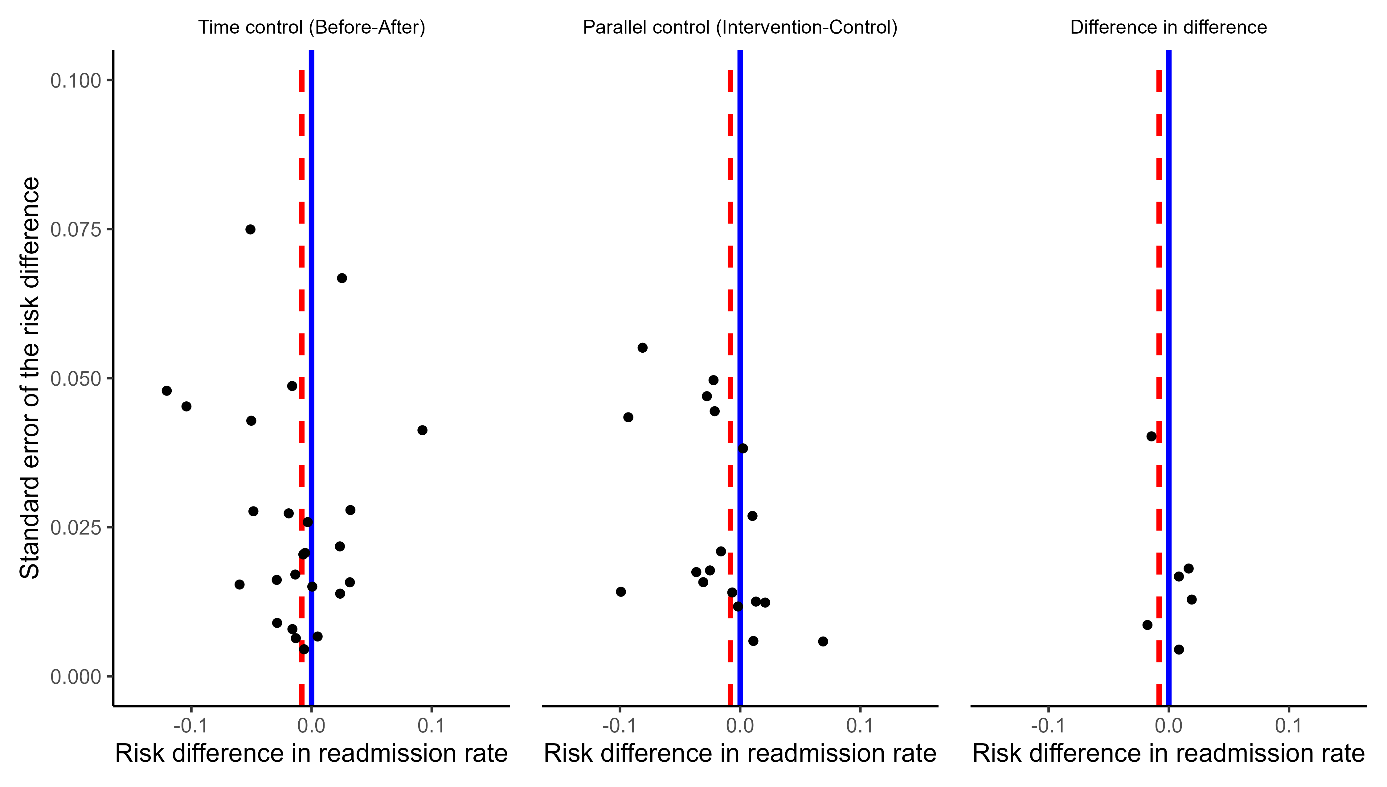


Figure S5: Funnel plot for readmission rate by study designs. Red dotted line: Random effect estimate, blue line: risk difference of 0


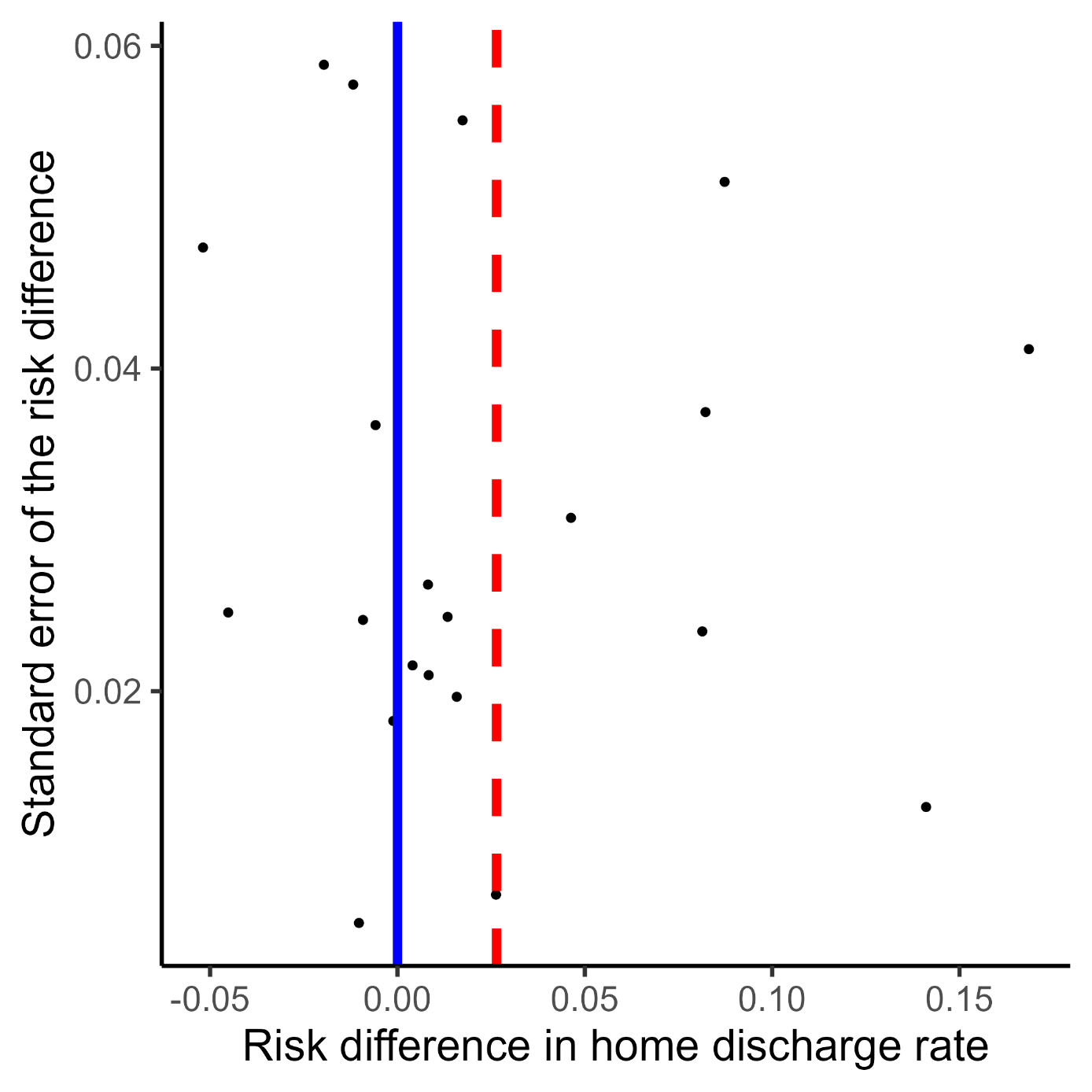


Figure S6: Funnel plot for home discharge rate. Red dotted line: Random effect estimate, blue line: risk difference of 0


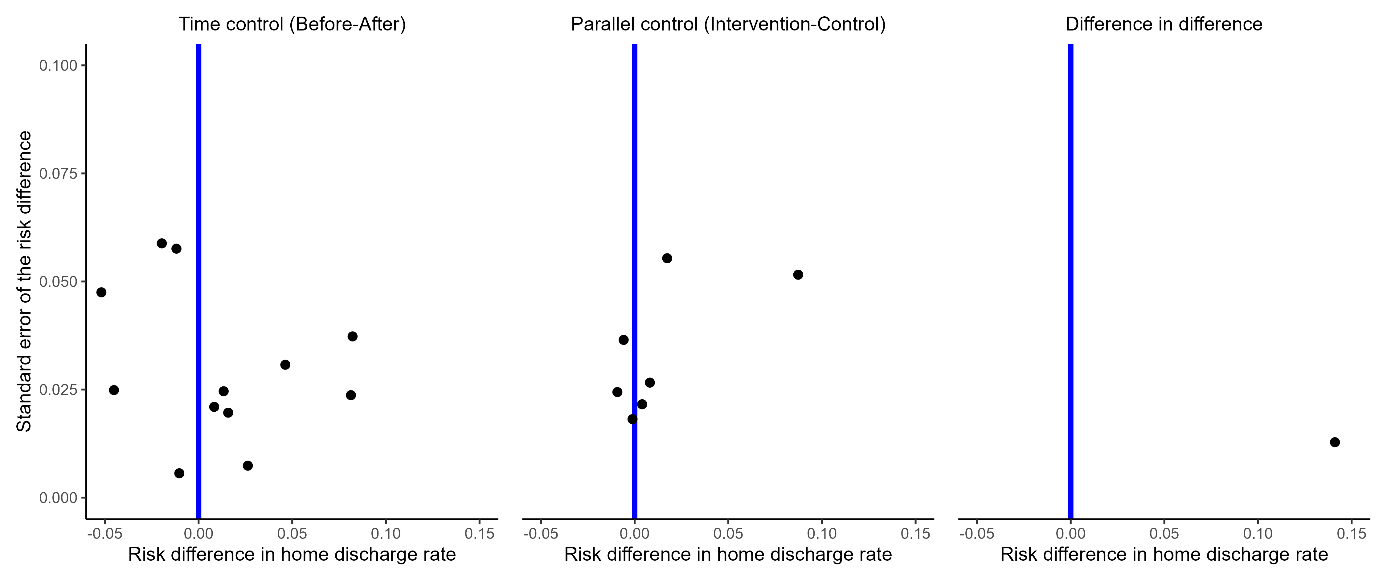


Figure S7: Funnel plot for home discharge rate by study designs. Blue line: risk difference of 0


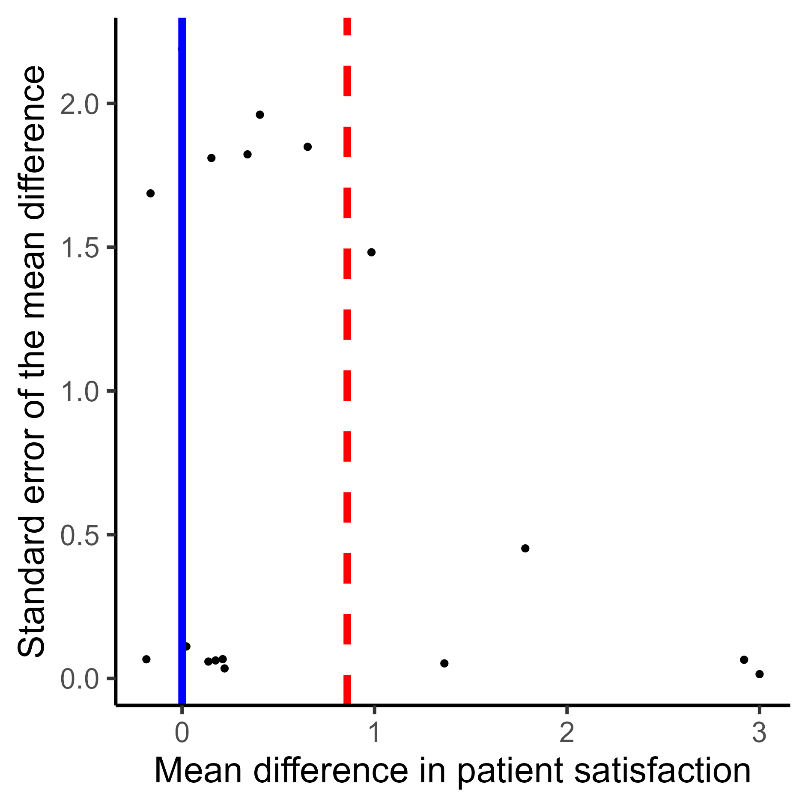


Figure S7: Funnel plot for studies reporting a patient satisfaction in mean. Red dotted line: Random effect estimate, blue line: mean difference of 0


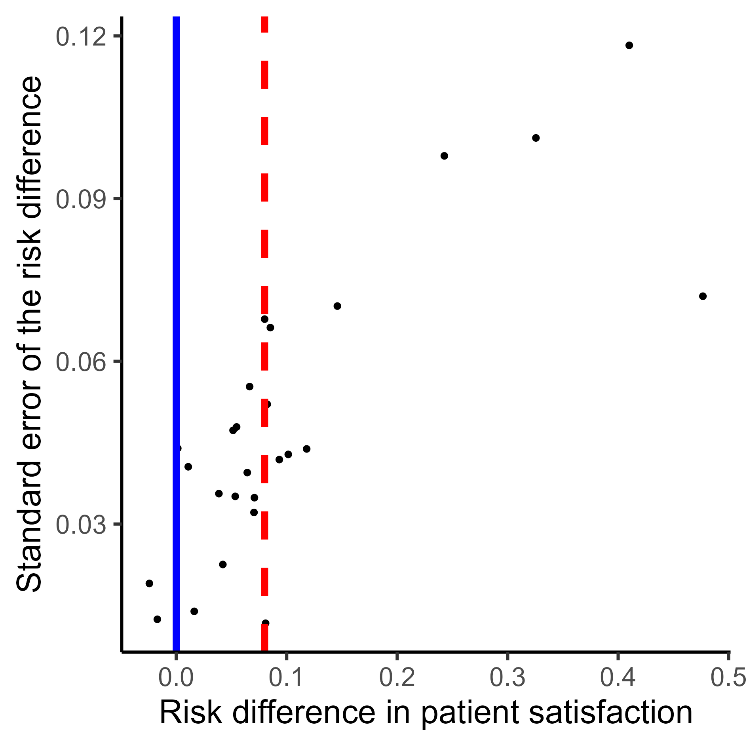


Figure S8: Funnel plot for studies reporting a patient satisfaction in proportion. Red dotted line: Random effect estimate, blue line: risk difference of 0

# Appendix 5: Flow charts by outcomes


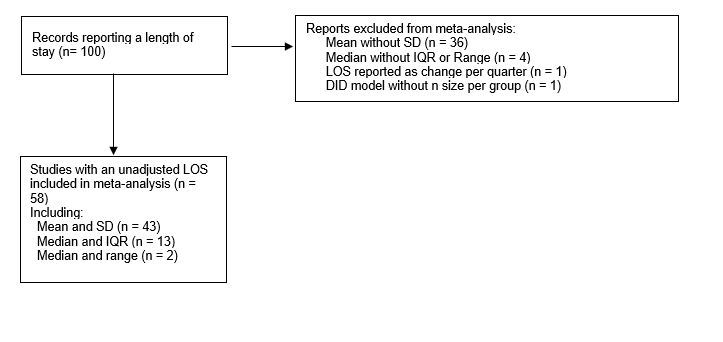


Figure S9: Flowchart of included studies for length of stay. SD = Standard Deviation, IQR = Interquartile Range, LOS = Length of Stay, DID = Difference-in-difference


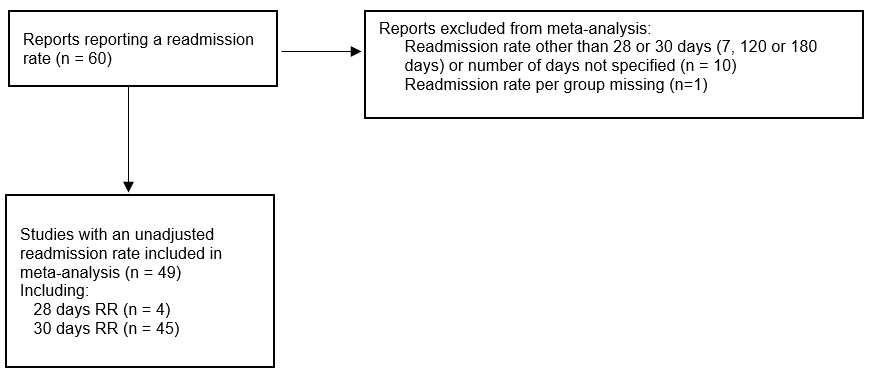


Figure S10: Flowchart of included studies for readmission rate. RR = Readmission rate


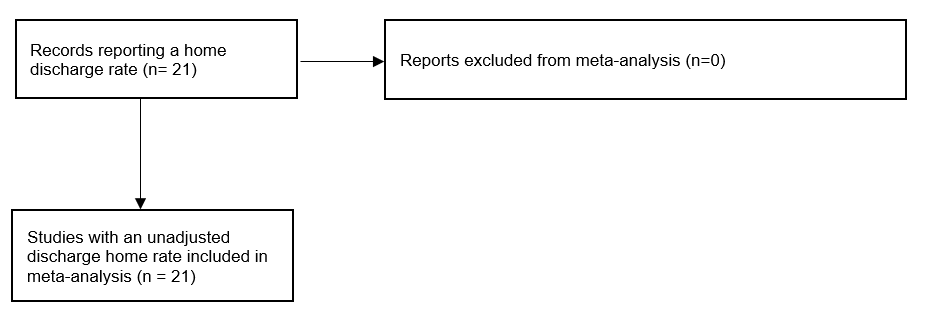


Figure S11: Flowchart of included studies for home discharge rate


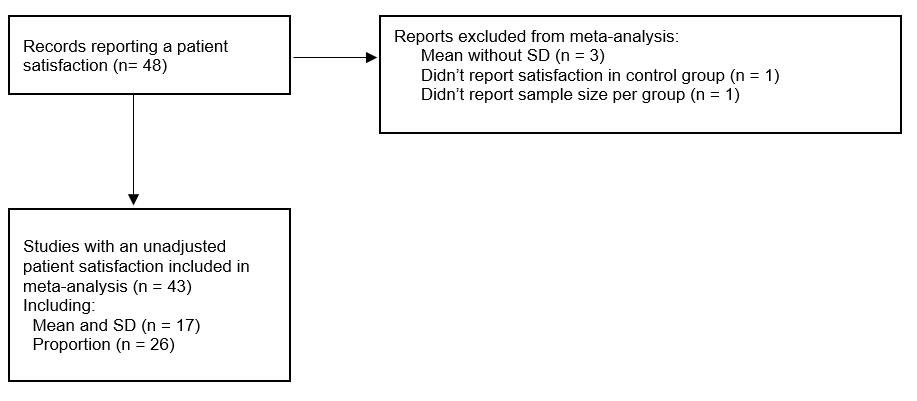


Figure S12: Flowchart of included studies for patient satisfaction

# Appendix 6: Forest plots by outcomes


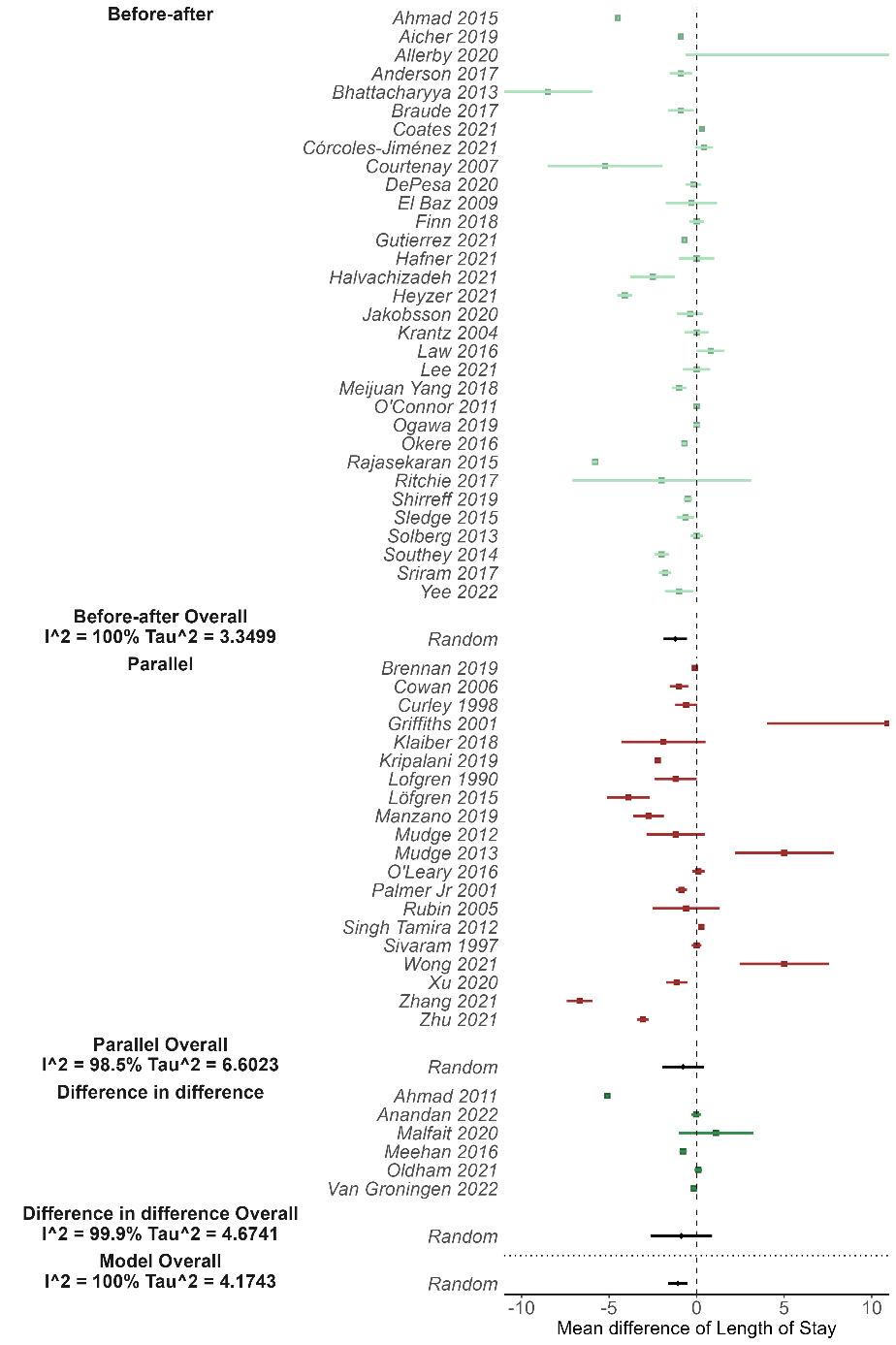

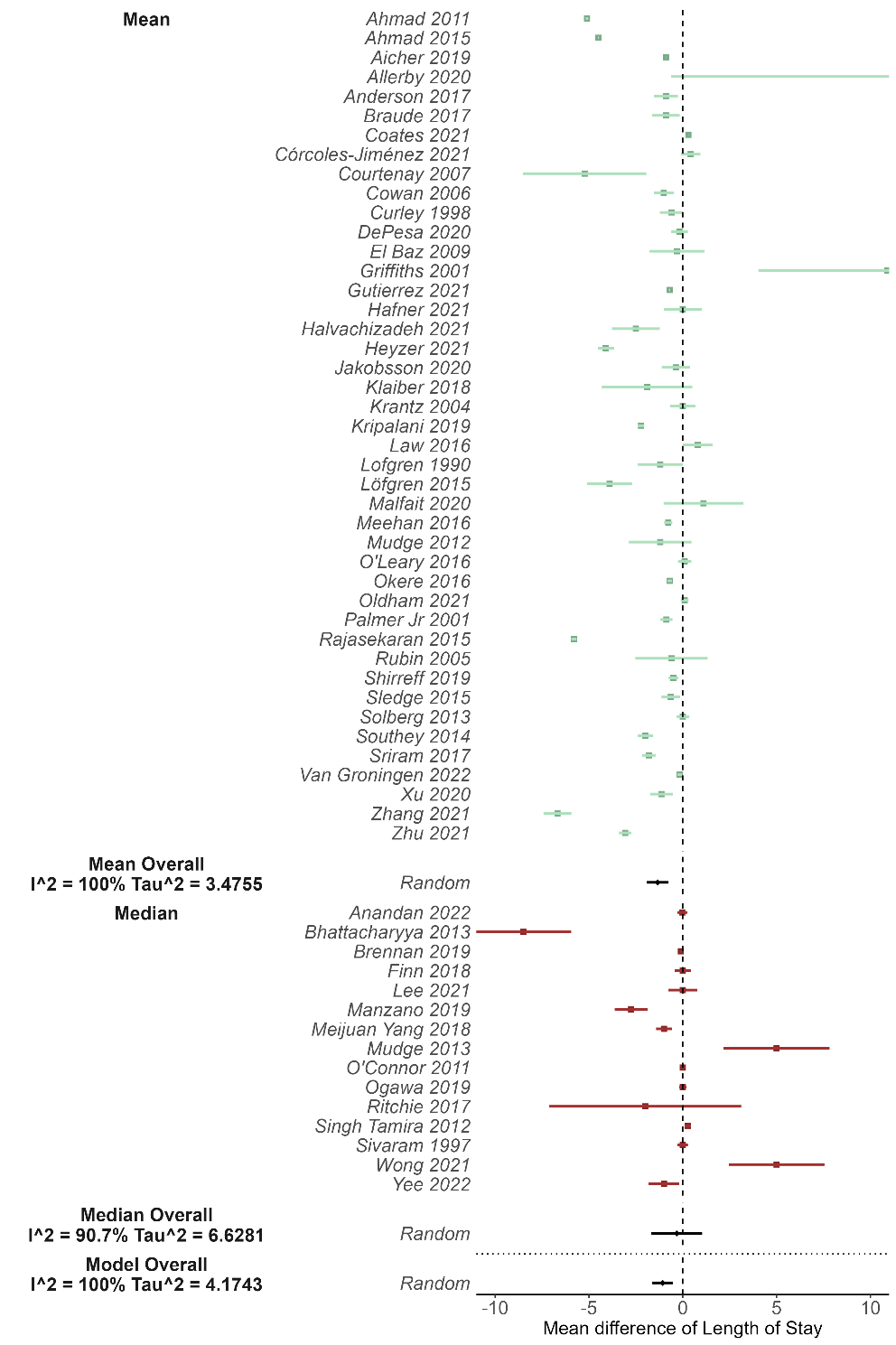


Figure S13: Forest Plot for the length of stay outcome stratified by study design and by modality of length of stay reporting (mean/median). NB: graphs were cropped from -10: +10 on the mean length of stay for readability. The only estimate not displayed is from Allerby 2020 and was +13.90 [-0.62: +28.42]


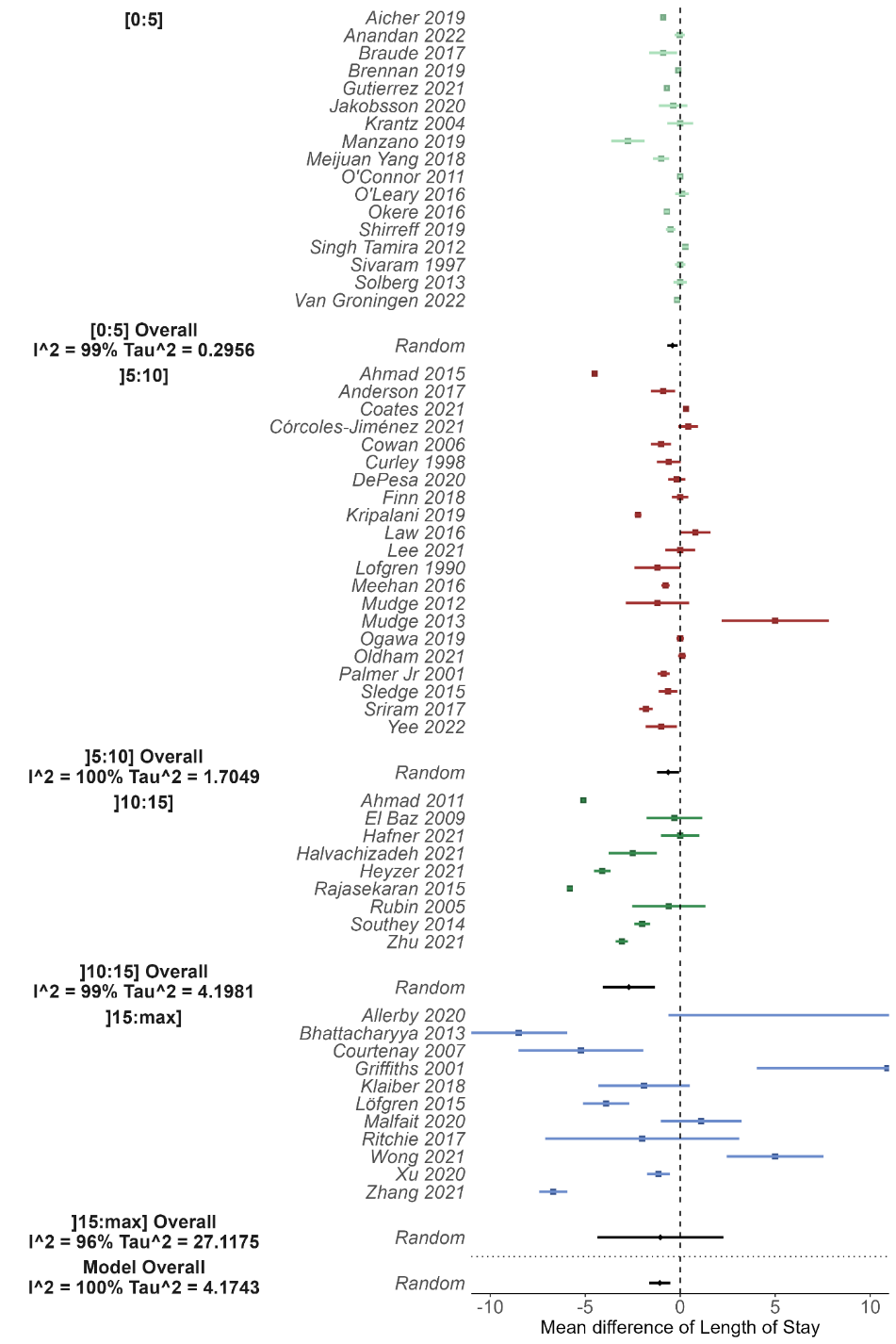


Figure S14: Forest Plot of mean difference in length of stay stratified by LOS prior to intervention. NB: graphs were cropped from -10: +10 for readability (The only mean difference not displayed is from Allerby 2020 and was +13.90 [-0.62: +28.42])


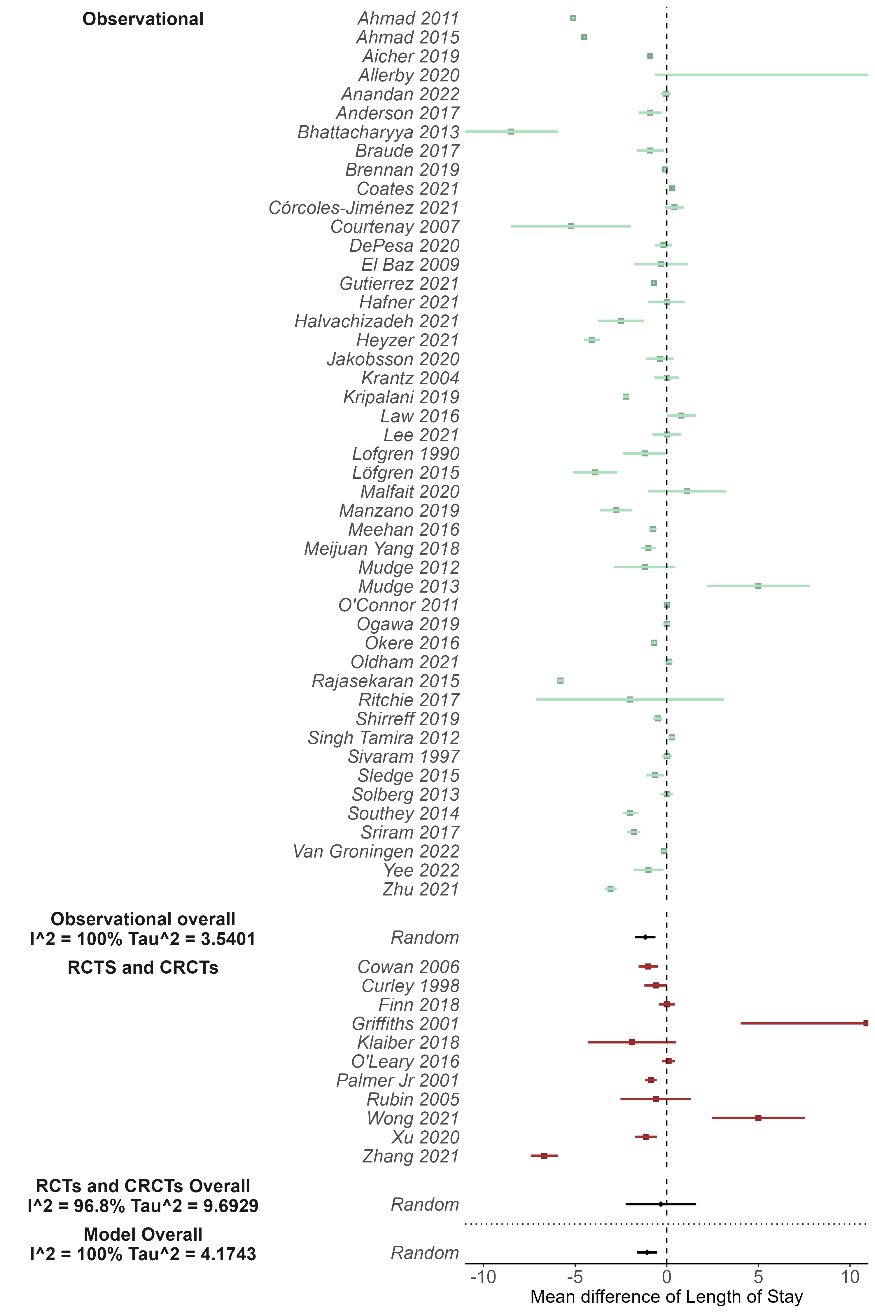


Figure S15: Forest Plot of mean difference in length of stay stratified by study design


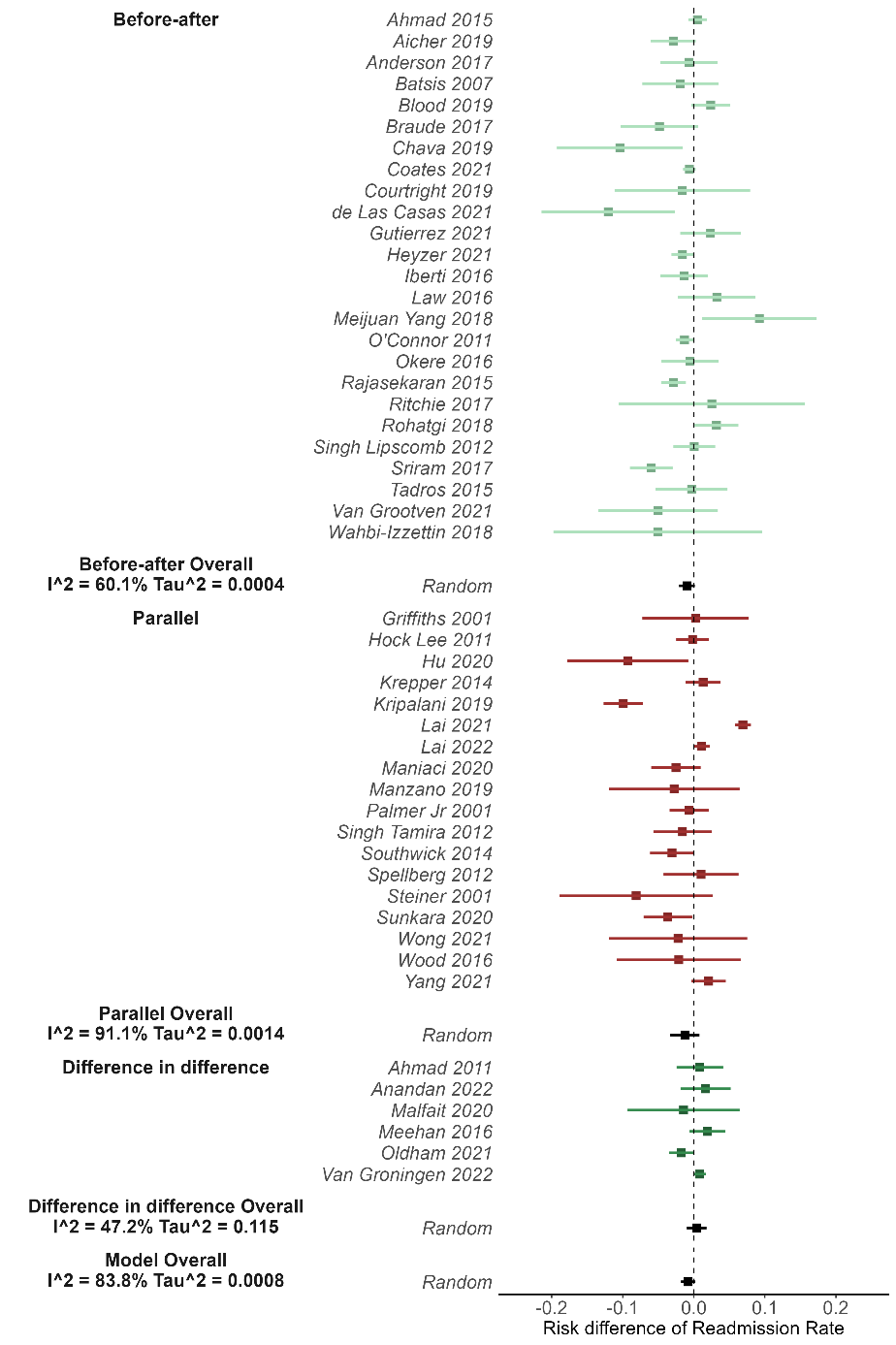


*Figure S16: Forest Plot of Readmission rate stratified by comparator type*


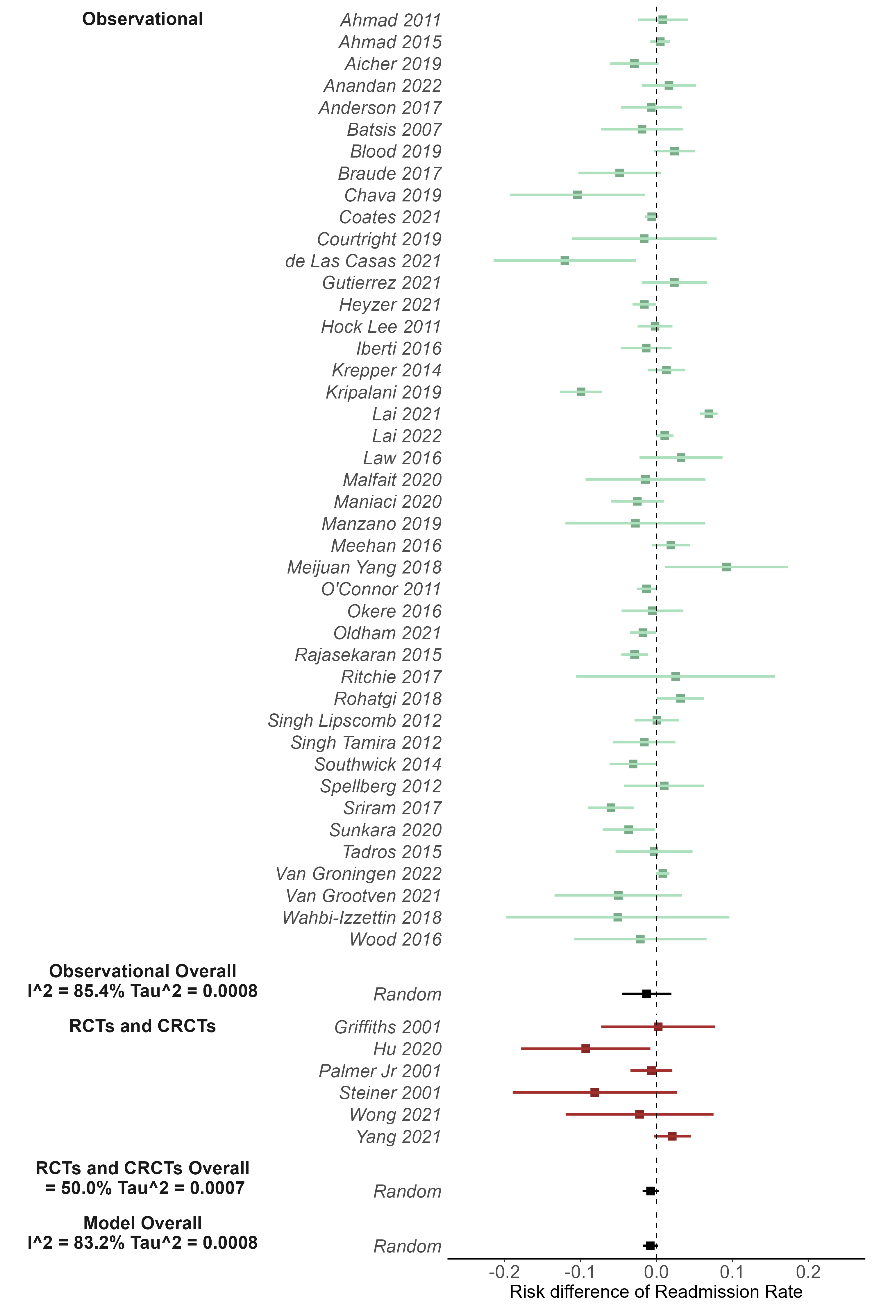


Figure S17: Forest plot of Readmission rate stratified by study design


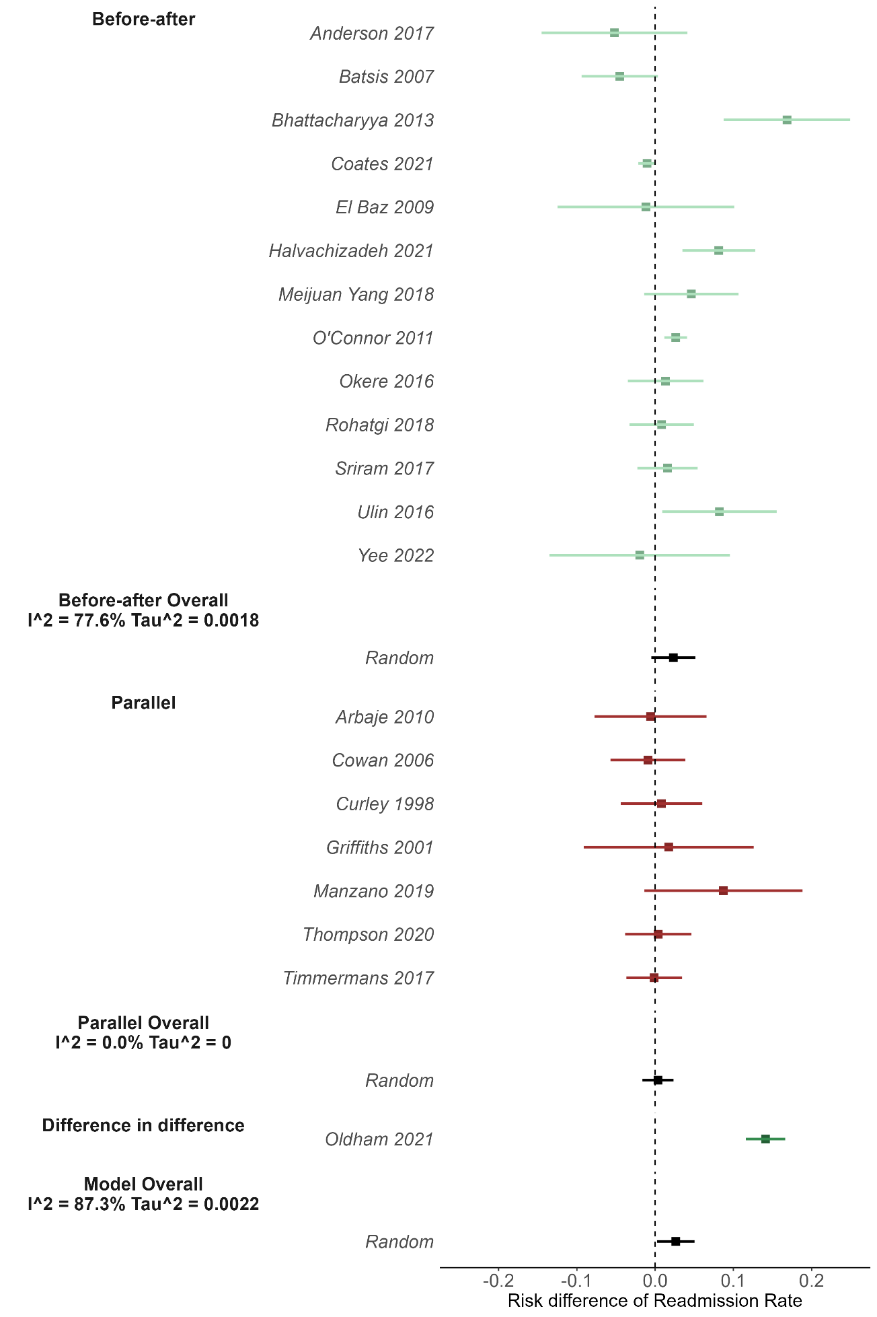

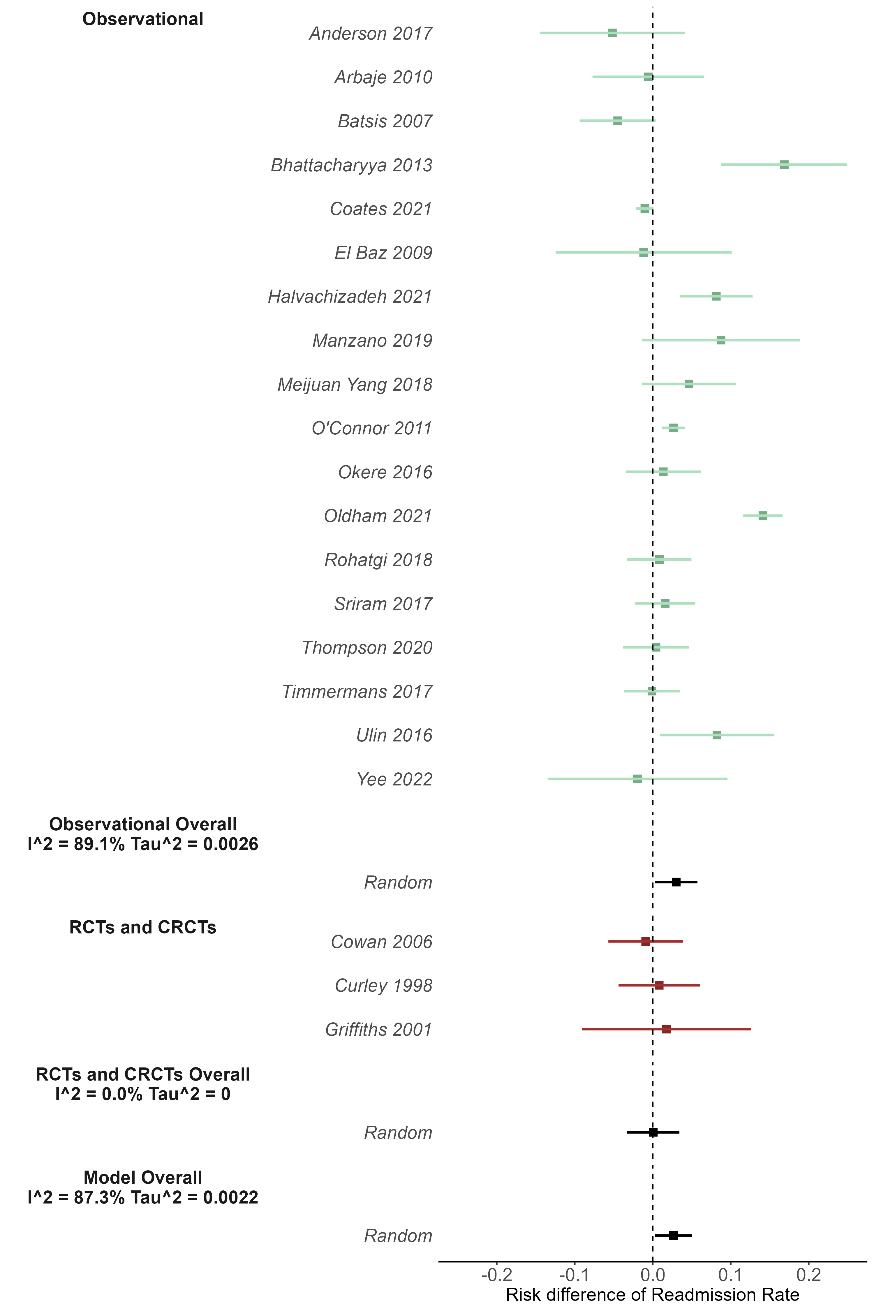


Figure S18: Forest Plot for the rate of home discharge with pooled effects by study design


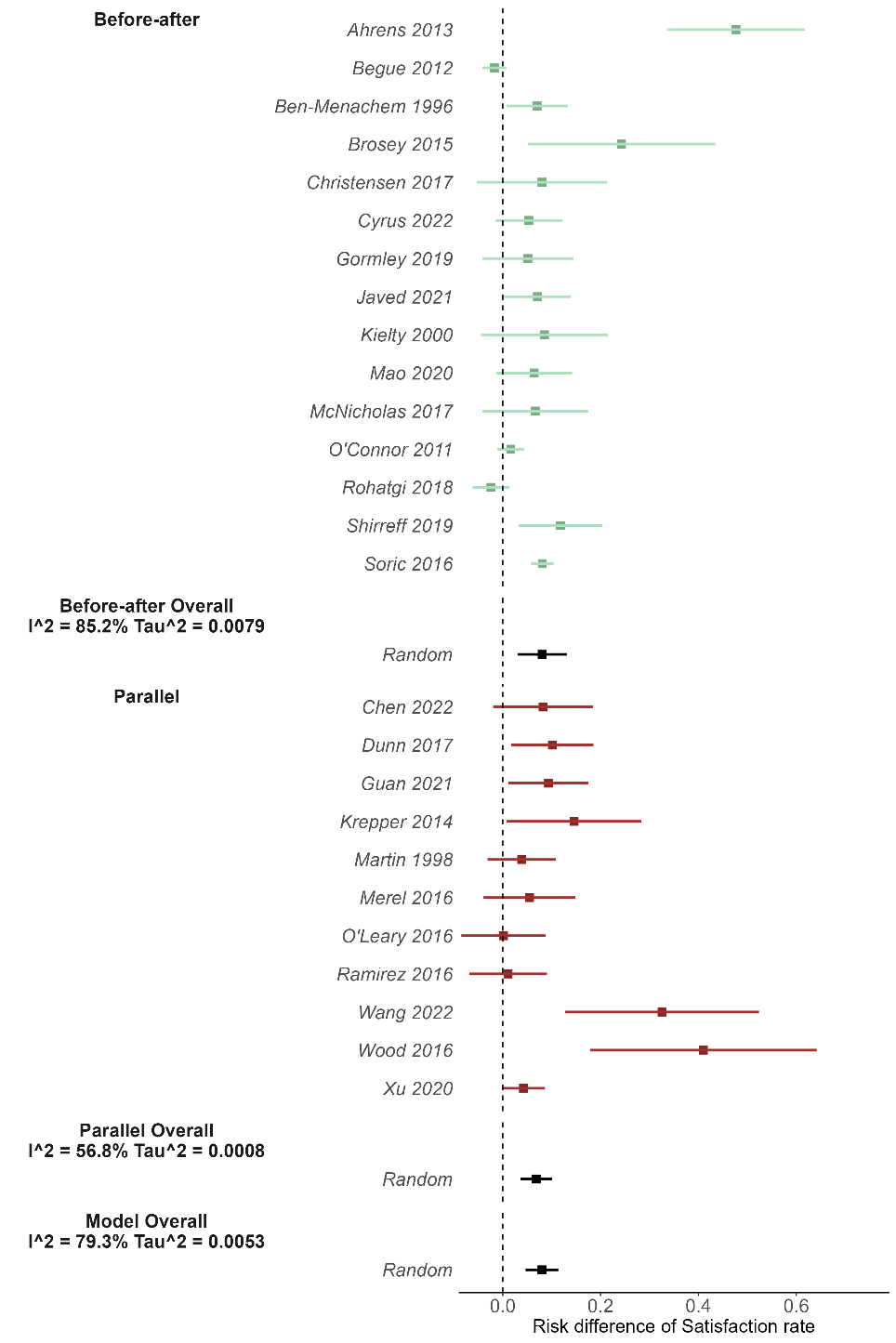

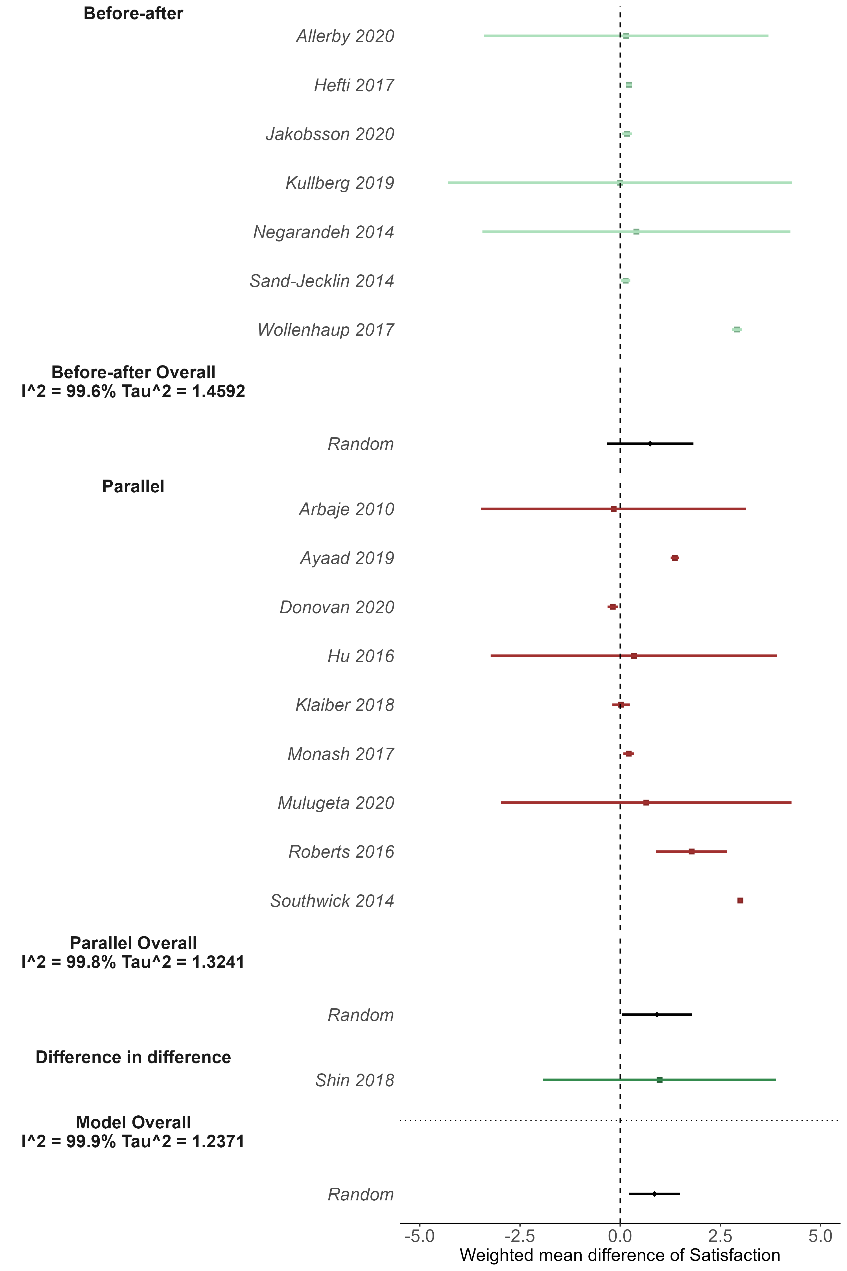


*Figure S19: Forest Plot for the patient satisfaction rate and mean with pooled effects by study design*


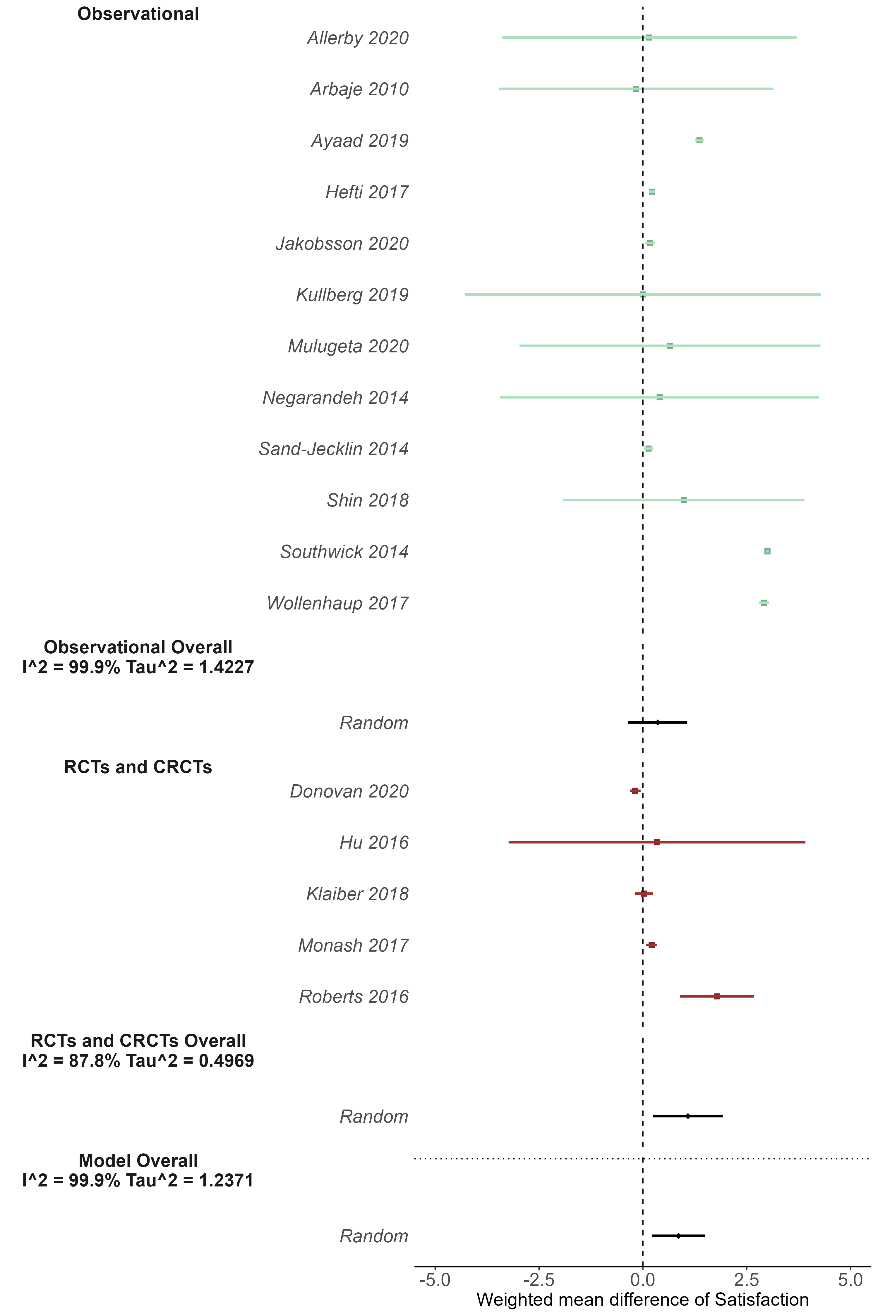

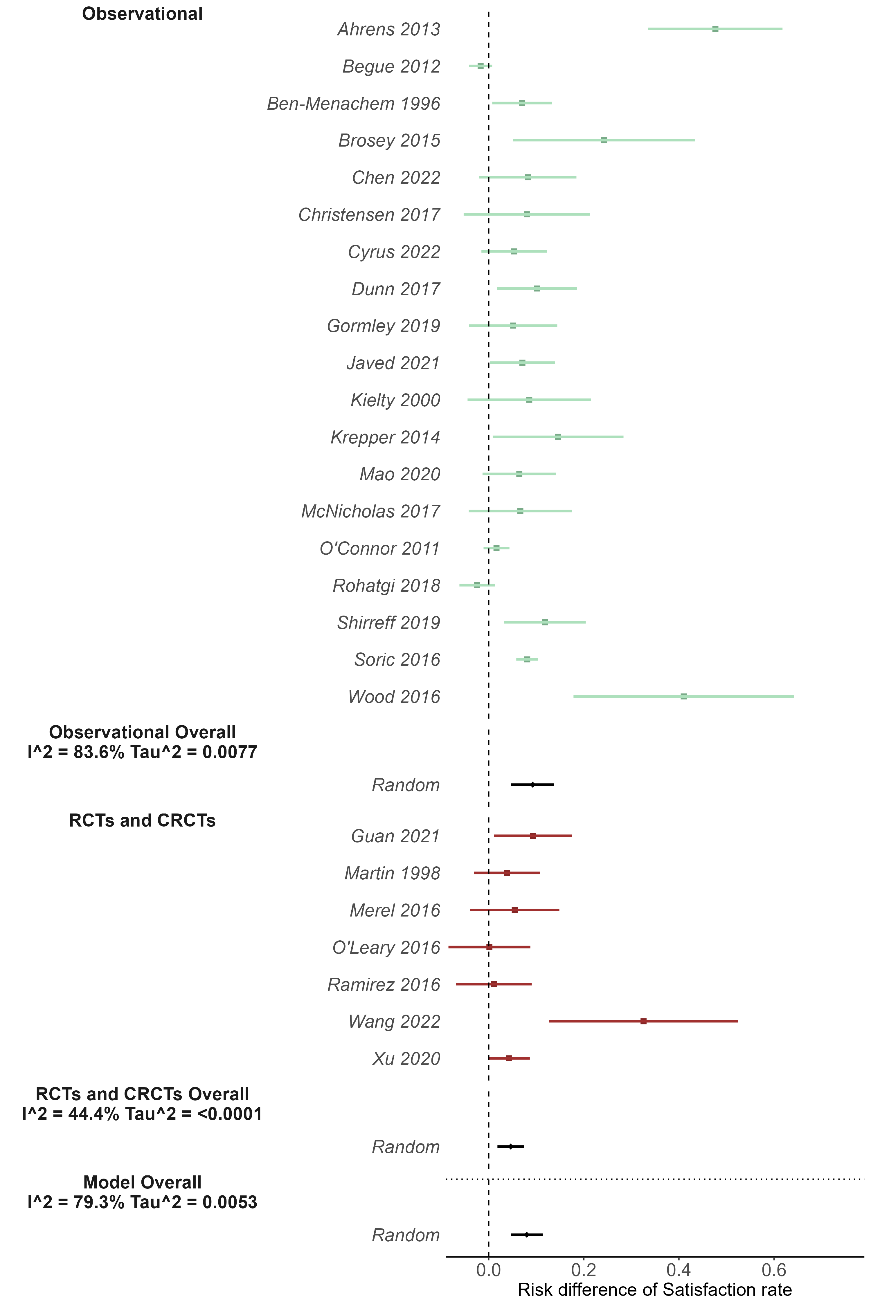


*Figure S20: Forest Plot for the patient satisfaction rate and mean with pooled effects by study design*

# Appendix 7: Risk of bias by domain for included articles

Table S4 Risk of Bias assessment via Robins I-Tool for Length of stay outcome

| Article Label | Design | Risk of Bias domain | | | | | | | |
| --- | --- | --- | --- | --- | --- | --- | --- | --- | --- |
|  |  | Confounder | Participation Selection | Classification | Protocol Deviation | Missing data | Outcome measurement | Selective reporting | Overall |
| Ahmad 2011 | Pure Observational | Serious/Critical | Low | Low | Low | Low | Low | Moderate | Critical |
| Ahmad 2015 | Pure Observational | Serious/Critical | Moderate | Low | Low | No information | Low | Moderate | Critical |
| Aicher 2019 | Pure Observational | Serious/Critical | Moderate | Low | Low | No information | Low | Moderate | Critical |
| Allerby 2020 | Pure Observational | Serious/Critical | Moderate | Low | Low | Low | Low | Moderate | Critical |
| Anandan 2022 | Pure Observational | Serious/Critical | Low | Low | No information | No information | Low | Moderate | Critical |
| Anderson 2017 | Pure Observational | Serious/Critical | Moderate | Low | Low | Low | Low | Moderate | Critical |
| Bhattacharyya 2013 | Pure Observational | Serious/Critical | Moderate | Low | Low | Low | Low | Moderate | Critical |
| Braude 2017 | Pure Observational | Moderate | Moderate | Low | Low | No information | Low | Moderate | Moderate |
| Brennan 2019 | Pure Observational | Serious/Critical | Low | Serious | Low | Low | Low | Serious | Critical |
| Coates 2021 | Pure Observational | Serious/Critical | Moderate | Low | Low | No information | Low | Moderate | Critical |
| Córcoles-Jiménez 2021 | Pure Observational | Serious/Critical | Moderate | Low | Low | Low | Low | Moderate | Critical |
| Courtenay 2007 | Pure Observational | Serious/Critical | Moderate | Low | Low | No information | Low | Moderate | Critical |
| DePesa 2020 | Pure Observational | Serious/Critical | Moderate | Low | Low | No information | Low | Moderate | Critical |
| El Baz 2009 | Matched | Serious/Critical | Moderate | Low | Low | Low | Serious | Moderate | Critical |
| Gutierrez 2021 | Pure Observational | Serious/Critical | Low | Low | Low | Low | Low | Moderate | Critical |
| Hafner 2021 | Pure Observational | Serious/Critical | Moderate | Low | No information | No information | Low | Moderate | Critical |
| Halvachizadeh 2021 | Pure Observational | Serious/Critical | Moderate | Low | No information | No information | Low | Moderate | Critical |
| Heyzer 2021 | Pure Observational | Serious/Critical | Moderate | Low | Low | Low | Low | Moderate | Critical |
| Jakobsson 2020 | Pure Observational | Serious/Critical | Moderate | Low | Low | Low | Serious | Serious | Critical |
| Krantz 2004 | Pure Observational | No information | Low | Low | Low | Low | Low | Moderate | Moderate |
| Kripalani 2019 | Pure Observational | No information | Moderate | Low | Low | No information | No information | Serious | Serious |
| Law 2016 | Pure Observational | Serious/Critical | Moderate | Low | Low | Low | Low | Moderate | Critical |
| Lee 2021 | Pure Observational | Serious/Critical | Low | Low | Low | No information | Low | Moderate | Critical |
| Lofgren 1990 | Pure Observational | Low | Low | Low | Low | Low | Low | Moderate | Moderate |
| Löfgren 2015 | Pure Observational | Serious/Critical | Low | Low | No information | No information | No information | Moderate | Critical |
| Malfait 2020 | Matched | Serious/Critical | Low | Low | Low | No information | Low | Moderate | Critical |
| Manzano 2019 | Pure Observational | Serious/Critical | Low | Low | Low | Low | Low | Moderate | Critical |
| Meehan 2016 | Pure Observational | Serious/Critical | Low | Low | Low | Low | Low | Moderate | Critical |
| Meijuan Yang 2018 | Pure Observational | Serious/Critical | Moderate | Low | Low | Low | Low | Moderate | Critical |
| Mudge 2012 | Pure Observational | Low | Low | Low | Low | Low | Low | Moderate | Moderate |
| Mudge 2013 | Pure Observational | Serious/Critical | Low | Serious | Low | Low | Low | Moderate | Critical |
| O'Connor 2011 | Pure Observational | Moderate | Moderate | Low | Low | No information | Low | Moderate | Moderate |
| Ogawa 2019 | Pure Observational | Moderate | Low | Low | No information | No information | Low | Serious | Serious |
| Okere 2016 | Matched | Moderate | Moderate | Low | Low | No information | Low | Moderate | Moderate |
| Oldham 2021 | Pure Observational | Moderate | Low | Low | Low | No information | Low | Moderate | Moderate |
| Rajasekaran 2015 | Pure Observational | Serious/Critical | Moderate | Low | Low | No information | Low | Moderate | Critical |
| Ritchie 2017 | Pure Observational | Serious/Critical | Low | Low | Low | Low | Low | Moderate | Critical |
| Shirreff 2019 | Pure Observational | Serious/Critical | Moderate | Low | Low | No information | Low | Moderate | Critical |
| Singh Tamira 2012 | Pure Observational | Serious/Critical | Low | Serious | Low | Low | Low | Moderate | Critical |
| Sivaram 1997 | Pure Observational | No information | No information | No information | No information | No information | No information | No information | No information |
| Sledge 2015 | Pure Observational | Serious/Critical | Moderate | Low | Low | No information | Low | Moderate | Critical |
| Solberg 2013 | Pure Observational | No information | No information | Low | No information | No information | No information | No information | No information |
| Southey 2014 | Pure Observational | No information | Moderate | Low | Low | No information | Low | Moderate | Moderate |
| Sriram 2017 | Pure Observational | Serious/Critical | Low | Low | Low | Low | Low | Moderate | Critical |
| Van Groningen 2022 | Pure Observational | Serious/Critical | Low | Low | Low | No information | Low | Moderate | Critical |
| Yee 2022 | Pure Observational | Serious/Critical | Moderate | Low | Low | No information | Low | Moderate | Critical |
| Zhu 2021 | Pure Observational | No information | No information | Low | No information | No information | No information | No information | No information |

Table S5 Risk of Bias assessment via Cochrane ROB-2 for Length of stay outcome

| Article Label | Design | Risk of Bias domain | | | | | |
| --- | --- | --- | --- | --- | --- | --- | --- |
|  |  | Randomization process | Timing of identification and recruitment of participant | Deviations from intended interventions | Missing outcome | Measurement of the outcome | Selection of the reported result |
| Griffiths 2001 | RCT | Low | Not relevant | Low | Low | Low | Some concerns |
| Palmer Jr 2001 | RCT | High | Not relevant | Low | Low | Low | Some concerns |
| Rubin 2005 | RCT | Some Concerns | Not relevant | Low | Low | Low | Some concerns |
| Wong 2021 | RCT | Low | Not relevant | Low | High | Low | Low |
| Zhang 2021 | RCT | Low | Not relevant | Low | Low | High | Some concerns |
| Cowan 2006 | CRCT | High | Low | Low | Low | Low | Some concerns |
| Curley 1998 | CRCT | High | Low | Low | Low | Low | Some concerns |
| Finn 2018 | CRCT | Low | Low | Low | Low | Low | Low |
| Klaiber 2018 | CRCT | Low | Low | Low | Low | Low | Low |
| O'Leary 2016 | CRCT | Some concerns | Low | Low | Low | Low | Some concerns |
| Xu 2020 | CRCT | Low | Low | Low | Low | Low | Some concerns |

Table S6 Risk of Bias assessment via Robins I-Tool for Readmission rate outcome

| Article Label | Design | Risk of Bias domain | | | | | | | |
| --- | --- | --- | --- | --- | --- | --- | --- | --- | --- |
|  |  | Confounder | Participation Selection | Classification | Protocol Deviation | Missing data | Outcome measurement | Selective reporting | Overall |
| Ahmad 2011 | Pure Observational | Serious/Critical | Low | Low | Low | Low | Low | Moderate | Critical |
| Ahmad 2015 | Pure Observational | Serious/Critical | Moderate | Low | Low | No information | Low | Moderate | Critical |
| Aicher 2019 | Pure Observational | Moderate | Moderate | Low | Low | No information | Low | Moderate | Moderate |
| Anandan 2022 | Pure Observational | Serious/Critical | Low | Low | No information | No information | Low | Moderate | Critical |
| Anderson 2017 | Pure Observational | Serious/Critical | Moderate | Low | Low | Low | Low | Moderate | Critical |
| Batsis 2007 | Pure Observational | Serious/Critical | Moderate | Low | Low | No information | Low | Moderate | Critical |
| Blood 2019 | Pure Observational | Serious/Critical | Low | Low | Low | Low | Low | Moderate | Critical |
| Braude 2017 | Pure Observational | Moderate | Moderate | Low | Low | No information | Low | Moderate | Moderate |
| Chava 2019 | Pure Observational | Serious/Critical | Moderate | Low | Low | No information | Low | Serious | Critical |
| Coates 2021 | Pure Observational | Serious/Critical | Moderate | Low | Low | No information | Low | Moderate | Critical |
| Courtright 2019 | Pure Observational | Moderate | No information | Low | Serious/Critical | Low | Low | No information | Critical |
| de Las Casas 2021 | Pure Observational | Serious/Critical | Moderate | Low | Low | Low | Low | Moderate | Critical |
| Gutierrez 2021 | Pure Observational | Serious/Critical | Low | Low | Low | Low | Low | Moderate | Critical |
| Heyzer 2021 | Pure Observational | Serious/Critical | Moderate | Low | Low | Low | Low | Moderate | Critical |
| Hock Lee 2011 | Pure Observational | Moderate | Moderate | Low | Low | No information | Low | Moderate | Moderate |
| Iberti 2016 | Pure Observational | Serious/Critical | Low | Low | Low | Low | Low | Moderate | Critical |
| Krepper 2014 | Pure Observational | Serious/Critical | Low | Low | Low | Low | Low | Moderate | Critical |
| Kripalani 2019 | Pure Observational | No information | Moderate | Low | Low | No information | No information | Serious | Serious |
| Lai 2021 | Pure Observational | Serious/Critical | Moderate | Low | No information | No information | Low | Moderate | Critical |
| Lai 2022 | Pure Observational | Serious/Critical | Low | Low | No information | No information | Low | Moderate | Critical |
| Law 2016 | Pure Observational | Serious/Critical | Moderate | Low | Low | Low | Low | Moderate | Critical |
| Malfait 2020 | Matched | Serious/Critical | Low | Low | Low | No information | Low | Moderate | Critical |
| Maniaci 2020 | Pure Observational | Serious/Critical | Low | Low | Low | Low | Serious | Moderate | Critical |
| Manzano 2019 | Pure Observational | Serious/Critical | Low | Low | Low | Low | Low | Moderate | Critical |
| Meehan 2016 | Pure Observational | Serious/Critical | Low | Low | Low | Low | Low | Moderate | Critical |
| Meijuan Yang 2018 | Pure Observational | Serious/Critical | Moderate | Low | Low | Low | Low | Moderate | Critical |
| O'Connor 2011 | Pure Observational | Moderate | Moderate | Low | Low | No information | Low | Moderate | Moderate |
| Okere 2016 | Matched | Moderate | Moderate | Low | Low | No information | Low | Moderate | Moderate |
| Oldham 2021 | Pure Observational | Moderate | Low | Low | Low | No information | Low | Moderate | Moderate |
| Rajasekaran 2015 | Pure Observational | Serious/Critical | Moderate | Low | Low | No information | Low | Moderate | Critical |
| Ritchie 2017 | Pure Observational | Serious/Critical | Low | Low | Low | Low | Low | Moderate | Critical |
| Rohatgi 2018 | Pure Observational | Moderate | Moderate | Low | Low | Low | Serious | Moderate | Serious |
| Singh Lipscomb 2012 | Pure Observational | No information | Low | Serious | Moderate | No information | No information | Moderate | Serious |
| Singh Tamira 2012 | Pure Observational | Serious/Critical | Low | Serious | Low | Low | Low | Moderate | Critical |
| Southwick 2014 | Pure Observational | Serious/Critical | Moderate | Low | Serious/Critical | No information | Low | Moderate | Critical |
| Spellberg 2012 | Pure Observational | Serious/Critical | Low | Low | Low | Low | Low | Moderate | Critical |
| Sriram 2017 | Pure Observational | Serious/Critical | Low | Low | Low | Low | Low | Moderate | Critical |
| Sunkara 2020 | Pure Observational | Moderate | Low | Serious | Low | Low | Serious | Moderate | Serious |
| Tadros 2015 | Pure Observational | Moderate | Moderate | Low | Low | Low | Low | Moderate | Moderate |
| Van Groningen 2022 | Pure Observational | Serious/Critical | Low | Low | Low | No information | Low | Moderate | Critical |
| Van Grootven 2021 | Pure Observational | Moderate | Moderate | Serious | Low | No information | Serious | Moderate | Serious |
| Wahbi-Izzettin 2018 | Pure Observational | Serious/Critical | Moderate | Low | No information | No information | Low | Moderate | Critical |
| Wood 2016 | Pure Observational | Serious/Critical | Low | Serious | Low | No information | Low | Moderate | Critical |

Table 7 Risk of Bias assessment via Cochrane ROB-2 for Readmission rate outcome

| Article Label | Design | Risk of Bias category | | | | | |
| --- | --- | --- | --- | --- | --- | --- | --- |
|  |  | Randomization process | Timing of identification and recruitment of participant | Deviations from intended interventions | Missing outcome | Measurement of the outcome | Selection of the reported result |
| Griffiths 2001 | RCT | Low | Not relevant | Low | Low | Low | Some concerns |
| Hu 2020 | RCT | Low | Not relevant | Some concerns | Low | Low | Low |
| Palmer Jr 2001 | RCT | High | Not relevant | Low | Low | Low | Some concerns |
| Steiner 2001 | RCT | High | Not relevant | Low | Low | Low | Some concerns |
| Wong 2021 | RCT | Low | Not relevant | Low | High | Low | Low |
| Yang 2021 | CRCT | Low | Low | Low | Low | Low | Low |

Table S8 Risk of Bias assessment via Robins I-Tool for Home discharge rate outcome

| Article Label | Design | Risk of Bias category | | | | | | | |
| --- | --- | --- | --- | --- | --- | --- | --- | --- | --- |
|  |  | Confounder | Participation | Classification | Protocol Deviation | Missing data | Outcome measurement | Selective reporting | Overall study |
| Anderson 2017 | Pure Observational | Serious/Critical | Moderate | Low | Low | Low | Low | Moderate | Critical |
| Arbaje 2010 | Pure Observational | Serious/Critical | Low | Serious | Low | Low | Low | Moderate | Critical |
| Batsis 2007 | Pure Observational | Serious/Critical | Moderate | Low | Low | No information | Low | Moderate | Critical |
| Bhattacharyya 2013 | Pure Observational | Serious/Critical | Moderate | Low | Low | Low | Low | Moderate | Critical |
| Coates 2021 | Pure Observational | Serious/Critical | Moderate | Low | Low | No information | Low | Moderate | Critical |
| El Baz 2009 | Matched | Serious/Critical | Moderate | Low | Low | Low | Serious | Moderate | Critical |
| Halvachizadeh 2021 | Pure Observational | Serious/Critical | Moderate | Low | No information | No information | Low | Moderate | Critical |
| Manzano 2019 | Pure Observational | Serious/Critical | Low | Low | Low | Low | Low | Moderate | Critical |
| Meijuan Yang 2018 | Pure Observational | Serious/Critical | Moderate | Low | Low | Low | Low | Moderate | Critical |
| O'Connor 2011 | Pure Observational | Serious/Critical | Moderate | Low | Low | Low | Serious | Moderate | Critical |
| Okere 2016 | Matched | Moderate | Moderate | Low | Low | No information | Low | Moderate | Moderate |
| Oldham 2021 | Pure Observational | Moderate | Low | Low | Low | No information | Low | Moderate | Moderate |
| Rohatgi 2018 | Pure Observational | Serious/Critical | Moderate | Low | Low | Low | Low | Moderate | Critical |
| Sriram 2017 | Pure Observational | Serious/Critical | Low | Low | Low | Low | Low | Moderate | Critical |
| Thompson 2020 | Pure Observational | Serious/Critical | Moderate | Low | No information | No information | Low | Moderate | Critical |
| Timmermans 2017 | Matched | Serious/Critical | Low | Low | Low | No information | Low | Moderate | Critical |
| Ulin 2016 | Pure Observational | Serious/Critical | Moderate | Serious | Serious/Critical | Low | Serious | Moderate | Critical |
| Yee 2022 | Pure Observational | Serious/Critical | Moderate | Low | Low | No information | Low | Moderate | Critical |

Table S9 Risk of Bias assessment via Cochrane ROB-2 for Home discharge rate outcome

| Article Label | Design | Risk of Bias category | | | | | |
| --- | --- | --- | --- | --- | --- | --- | --- |
|  |  | Randomization process | Timing of identification and recruitment of participant | Deviations from intended interventions | Missing outcome | Measurement of the outcome | Selection of the reported result |
| Griffiths 2001 | RCT | Low | Not Relevant | Low | Low | Low | Some concerns |
| Cowan 2006 | CRCT | High | Low | Low | Low | Low | Some concerns |
| Curley 1998 | CRCT | High | Low | Low | Low | Low | Some concerns |

Table S8 Risk of Bias assessment via Robins I-Tool for Patient Satisfaction outcome

| Article Label | Design | Risk of Bias category | | | | | | | |
| --- | --- | --- | --- | --- | --- | --- | --- | --- | --- |
|  |  | Confounder | Participation Selection | Classification | Protocol Deviation | Missing data | Outcome measurement | Selective reporting | Overall study |
| Ahrens 2013 | Pure Observational | Low | Moderate | Low | Low | No information | Low | Moderate | Moderate |
| Allerby 2020 | Pure Observational | Serious/Critical | Moderate | Low | Low | Low | Low | Moderate | Critical |
| Arbaje 2010 | Pure Observational | Serious/Critical | Low | Low | Low | Moderate/Serious | Low | Moderate | Critical |
| Ayaad 2019 | Pure Observational | Serious/Critical | Low | Low | Low | Low | Low | Moderate | Critical |
| Begue 2012 | Pure Observational | Serious/Critical | Low | Low | Low | No information | Low | Moderate | Critical |
| Ben-Menachem 1996 | Pure Observational | Serious/Critical | Moderate | Low | Low | No information | Low | Moderate | Critical |
| Brosey 2015 | Pure Observational | Serious/Critical | Low | Low | Low | No information | Low | Moderate | Critical |
| Chen 2022 | Pure Observational | Low | Low | Low | Low | Low | No information | Moderate | Moderate |
| Christensen 2017 | Pure Observational | Serious/Critical | Moderate | Low | Low | Low | Low | Moderate | Critical |
| Cyrus 2022 | Pure Observational | Serious/Critical | Moderate | Low | Low | No information | Low | Moderate | Critical |
| Dunn 2017 | Pure Observational | Serious/Critical | Low | Low | Low | No information | Low | Serious | Critical |
| Gormley 2019 | Pure Observational | Serious/Critical | Moderate | Low | Low | No information | Serious | Moderate | Critical |
| Hefti 2017 | Pure Observational | Serious/Critical | Moderate | Low | Low | Low | Low | Moderate | Critical |
| Jakobsson 2020 | Pure Observational | Serious/Critical | Moderate | Low | Low | Low | Serious | Serious | Critical |
| Javed 2021 | Pure Observational | Serious/Critical | Moderate | Low | No information | No information | Serious | Moderate | Critical |
| Kielty 2000 | Pure Observational | Low | Moderate | Low | Low | No information | Low | Moderate | Moderate |
| Krepper 2014 | Pure Observational | Serious/Critical | Low | Low | Low | Low | Low | Moderate | Critical |
| Kullberg 2019 | Pure Observational | Low | Low | Low | Low | Low | Low | Moderate | Moderate |
| Mao 2020 | Pure Observational | Serious/Critical | Moderate | Low | Low | No information | No information | Moderate | Critical |
| McNicholas 2017 | Pure Observational | Serious/Critical | Moderate | Low | Low | Low | Serious | Moderate | Critical |
| Mulugeta 2020 | Pure Observational | Serious/Critical | Low | Serious | Low | No information | Serious | Moderate | Critical |
| Negarandeh 2014 | Pure Observational | Serious/Critical | Moderate | Low | Low | Low | Low | Moderate | Critical |
| O'Connor 2011 | Pure Observational | Serious/Critical | Moderate | Low | Low | Low | Serious | Moderate | Critical |
| Rohatgi 2018 | Pure Observational | Moderate | Moderate | Low | Low | Low | Serious | Moderate | Serious |
| Sand-Jecklin 2014 | Pure Observational | Serious/Critical | Moderate | Low | Serious/Critical | Low | Low | Serious | Critical |
| Shin 2018 | Pure Observational | Serious/Critical | Low | Low | Low | No information | No information | Moderate | Critical |
| Shirreff 2019 | Pure Observational | Serious/Critical | Moderate | Low | Low | No information | Low | Moderate | Critical |
| Soric 2016 | Pure Observational | Serious/Critical | Moderate | Low | Low | No information | Low | Moderate | Critical |
| Southwick 2014 | Pure Observational | Serious/Critical | Moderate | Low | Serious/Critical | No information | Low | Moderate | Critical |
| Wollenhaup 2017 | Pure Observational | No information | No information | Low | No information | No information | No information | No information | No information |
| Wood 2016 | Pure Observational | Serious/Critical | Low | Serious | Low | No information | Low | Moderate | Critical |

Table S10 Risk of Bias assessment via Cochrane ROB-2 for Patient Satisfaction outcome

| Article Label | Design | Risk of Bias category | | | | | |
| --- | --- | --- | --- | --- | --- | --- | --- |
|  |  | Randomization process | Timing of identification and recruitment of participant | Deviations from intended interventions | Missing outcome | Measurement of the outcome | Selection of the reported result |
| Guan 2021 | RCT | Some Concerns | Not relevant | Some concerns | Low | Low | Some concerns |
| Hu 2016 | RCT | Low | Not relevant | Low | Low | High | Some concerns |
| Martin 1998 | RCT | Some Concerns | Not relevant | Low | Low | High | Some concerns |
| Ramirez 2016 | RCT | Low | Not relevant | Low | Low | High | Some concerns |
| Roberts 2016 | RCT | Low | Not relevant | Low | Low | High | Low |
| Wang 2022 | RCT | Low | Not relevant | Low | Low | High | High |
| Donovan 2020 | CRCT | High | Low | Low | Low | Low | Some concerns |
| Klaiber 2018 | CRCT | Low | Low | Low | Low | High | Low |
| Merel 2016 | CRCT | High | Low | Low | Low | Low | Some concerns |
| Monash 2017 | CRCT | High | Low | Low | Low | Low | Low |
| O'Leary 2016 | CRCT | Some concerns | Low | Low | Low | Low | Some concerns |
| Xu 2020 | CRCT | Low | Low | Low | Low | Low | Some concerns |
